# Metabolic disassembler for understanding and predicting the biosynthetic units of natural products

**DOI:** 10.1186/s12859-019-3183-9

**Published:** 2019-12-23

**Authors:** Kohei Amano, Tsubasa Matsumoto, Kenichi Tanaka, Kimito Funatsu, Masaaki Kotera

**Affiliations:** 1 School of Life Science and Technology, Tokyo Institute of Technology, 2-12-1 Ookayama, Meguro-ku, Tokyo, 152-8550 Japan; 2 Department of Chemical System Engineering, School of Engineering, The University of Tokyo, 7-3-1 Hongo, Bunkyo-ku, Tokyo, 113-8656 Japan

**Keywords:** Natural products, Biosynthetic pathway, Starting material

## Abstract

**Background:**

Natural products are the source of various functional materials such as medicines, and understanding their biosynthetic pathways can provide information that is helpful for their effective production through the synthetic biology approach. A number of studies have aimed to predict biosynthetic pathways from their chemical structures in a retrosynthesis manner; however, sometimes the calculation finishes without reaching the starting material from the target molecule. In order to address this problem, the method to find suitable starting materials is required.

**Results:**

In this study, we developed a predictive workflow named the Metabolic Disassembler that automatically disassembles the target molecule structure into relevant biosynthetic units (BUs), which are the substructures that correspond to the starting materials in the biosynthesis pathway. This workflow uses a biosynthetic unit library (BUL), which contains starting materials, key intermediates, and their derivatives. We obtained the starting materials from the KEGG PATHWAY database, and 765 BUs were registered in the BUL. We then examined the proposed workflow to optimize the combination of the BUs. To evaluate the performance of the proposed Metabolic Disassembler workflow, we used 943 molecules that are included in the secondary metabolism maps of KEGG PATHWAY. About 95.8% of them (903 molecules) were correctly disassembled by our proposed workflow. For comparison, we also implemented a genetic algorithm-based workflow, and found that the accuracy was only about 52.0%. In addition, for 90.7% of molecules, our workflow finished the calculation within one minute.

**Conclusions:**

The Metabolic Disassembler enabled the effective disassembly of natural products in terms of both correctness and computational time. It also outputs automatically highlighted color-coded substructures corresponding to the BUs to help users understand the calculation results. The users do not have to specify starting molecules in advance, and can input any target molecule, even if it is not in databases. Our workflow will be very useful for understanding and predicting the biosynthesis of natural products.

## Background

Plants biosynthesize various natural products that protect them from being eaten by herbivores. Herbivore insects can detoxify these phytotoxic molecules and can biosynthesize various molecules including pheromones. Fungi are also a source of valuable natural products such as antibiotics. Secondary metabolites are species-specific natural products that are not directly involved in the species survival. Secondary metabolites and their derivatives are used widely in medicines, cosmetics, and agriculture. For example, penicillin, which was discovered by Fleming in 1928 [1], was widely used as an antibiotic against infectious diseases, and avermectin, which was discovered by Omura in 1979 [2], is widely used as an anti-parasite drug for pets.

Identifying the biosynthetic route of natural products is not only of pharmacological interest, but is also required by engineers and biologists who use synthetic biology techniques [3–8]. Synthetic biologists aim to synthesize desired molecules enzymatically by introducing foreign genes into an organism such as *Saccharomyces cerevisiae*. The biosynthesis need not necessarily mimic the actual biosynthetic pathway of the target molecule, but knowing the actual metabolic pathway can be very useful when constructing the pathway in a model organism.

However, although the chemical structures of a large number of metabolites are known, the metabolic pathways that lead to their synthesis are not yet known. Moreover, the recent development of high-throughput sequencers and metabolome analysis technology has revealed a large number of natural products for which the biosynthetic metabolic pathways are unknown. Currently, the identification of relevant enzymes in a putative pathway is carried out by trial and error based on knowledge and experience; therefore, an information science-based approach is highly desirable.

A large variety of natural products are produced from a limited variety of starting materials [9]. In this paper, we define a biosynthetic unit (BU) as a chemical substructure that is part of the starting material. Natural products can be disassembled into the same BUs or into several different BUs to help elucidate their biosynthetic pathways.

In organic synthesis, retrosynthetic analysis is used to design the synthetic route of a target molecule. The retrosynthetic analysis method uses the target molecule and constructs the route backward from the target molecule to the starting material. A similar approach can be applied to metabolic pathway prediction; however, the number of iterations required to generate all virtual reactions can easily explode, making it difficult to find an optimal solution in a finite time. In a previous study, machine learning was applied to decide whether a pair of metabolite molecules could form an enzymatic reaction, thereby showing the potential to predict the metabolic pathway [10]. Another previous study [11] is based on similarity search to find potential precursors, i.e., the compounds that are possibly reachable in artificially designed pathway in one step. However, these approaches only deal with one-step reactions in the metabolic pathways, and therefore do not consider the putative intermediates between the target molecule and the starting material. In other studies, retrosynthetic approach often failed to reach the starting material [3–8, 12]. To use the retrosynthetic approach for natural product biosynthesis, it is important to develop a workflow that can identify the appropriate starting materials.

In this study, we developed a workflow named the Metabolic Disassembler to automatically identify the BUs that corresponded to the starting materials of a given natural product. One of the important characteristics is that our method “disassembles” a given molecule into substructures that imply the starting materials. The aim was to provide support for identifying biosynthesis pathways. We evaluated the performance of the Metabolic Disassembler using datasets of secondary metabolites acquired from the Kyoto Encyclopedia of Genes and Genomes (KEGG) database [13], and showed the effectiveness of the workflow in terms of both accuracy and computational time. The users of the Metabolic Disassembler do not have to specify starting molecules in advance, and can input any target molecule, even if it is not in databases.

## Methods

### Chemical structures of natural products

The goal of the Metabolic Disassembler is not to construct artificial pathways, but is to know the actual metabolic pathways of natural products. In order to achieve our goal, compounds and reactions in primary (central) metabolism are not needed. Rather, they are a hindrance to the calculation. Also, if a secondary metabolite is not associated with known biosynthetic pathway, no one would estimate if the prediction is true. Therefore, we need to collect secondary metabolites for which biosynthetic pathways are already known. We surveyed databases including MetaCyc [14] and Rhea [15], and concluded that KEGG [13] (as of November 2018) is the one that stores necessary and sufficient amount of information.

In KEGG, metabolic compounds are given identifiers that contain the letter “C” and a five-digit number (e.g., “C00078” for L-tryptophan). We also used KEGG PATHWAY secondary metabolism maps to evaluate the accuracy of disassembly. In KEGG PATHWAY, secondary metabolism is classified as 1.9 “Metabolism of terpenoids and polyketides” (hereafter referred to as the 1.9 class) and 1.10 “Biosynthesis of other secondary metabolites” (hereafter referred to as the 1.10 class). The 1.9 class contains a group of molecules that are biosynthesized through mevalonate or non-mevalonate pathways, and BUs in these pathways are mostly limited to C_2_ and C_5_ units. This means that it is not difficult to identify these BUs because they are already quite obvious, and it is more important to analyze how the BUs form complex rings. Therefore, in this study, we focused on the 1.10 class, which contains 1111 molecules in 28 metabolic pathway maps. Molecules that had an “R” group, indicating they contained undefined structures, caused errors when using RDKit [16], and therefore were not used. The remaining 943 molecules were used in this study. Note that we used KEGG just because it covers all known pathways, and used KEGG PATHWAY just to test the performance. We designed the Metabolic Disassembler so that the users can input any molecule even if it is not found in any database. Note again that we did not focus on the primary (central) metabolites, because all secondary metabolites originate from the starting compounds in the secondary pathway maps. Therefore, in order to find the starting materials of unknown secondary metabolites, it is more practical to use them, rather than the primary (central) metabolites.

### Chemical structure manipulation and computing environment

We implemented our application software using Python programming language (version 3.6.6) on Anaconda3 (version 5.3.0) [17], NetworkX (version 2.2) [18], RDKit (version 2018.09.1.0) [16], Pycairo (version 1.18.0) [19], and CairoSVG (version 2.2.1) [20]. NetworkX is a graph calculation library that we used to produce the chemical structure graphs. RDKit is used widely in chemoinformatics, and we used it when retrieving chemical structures and conducting maximum common substructure searches. Pycairo and CairoSVG were used to render images of the molecules. The chemical structure of a molecule was represented as a chemical graph with atoms as nodes and chemical bonds as edges. Chemical structures described in MDL Molfile V2000 [21] were retrieved using RDKit and converted to chemical graphs using NetworkX. All calculations were conducted on a computer with an Intel Core i7-9700K 3.6 GHz CPU and 16 GB RAM in Windows 10 OS.

### Generating a biosynthetic unit library (BUL)

Some minimal units for the biosynthesis of natural products are known, including the acetate-C_2_ unit in the malonic acid pathway and the C_5_ unit in the isoprenoid pathway. The goal of this study was to identify the starting materials and to provide information that can be used to predict the biosynthesis pathway; therefore, it is more useful practically to deal with substructures that are larger than the minimal units, which correspond to the metabolites located near the boundary area of primary and secondary metabolism. We defined three types of biosynthetic units (BUs), basic BUs (BBUs), derivative BUs (DBUs), and preferential BUs (PBUs), and stored them in a biosynthetic unit library (BUL).

**Fig. 1:**
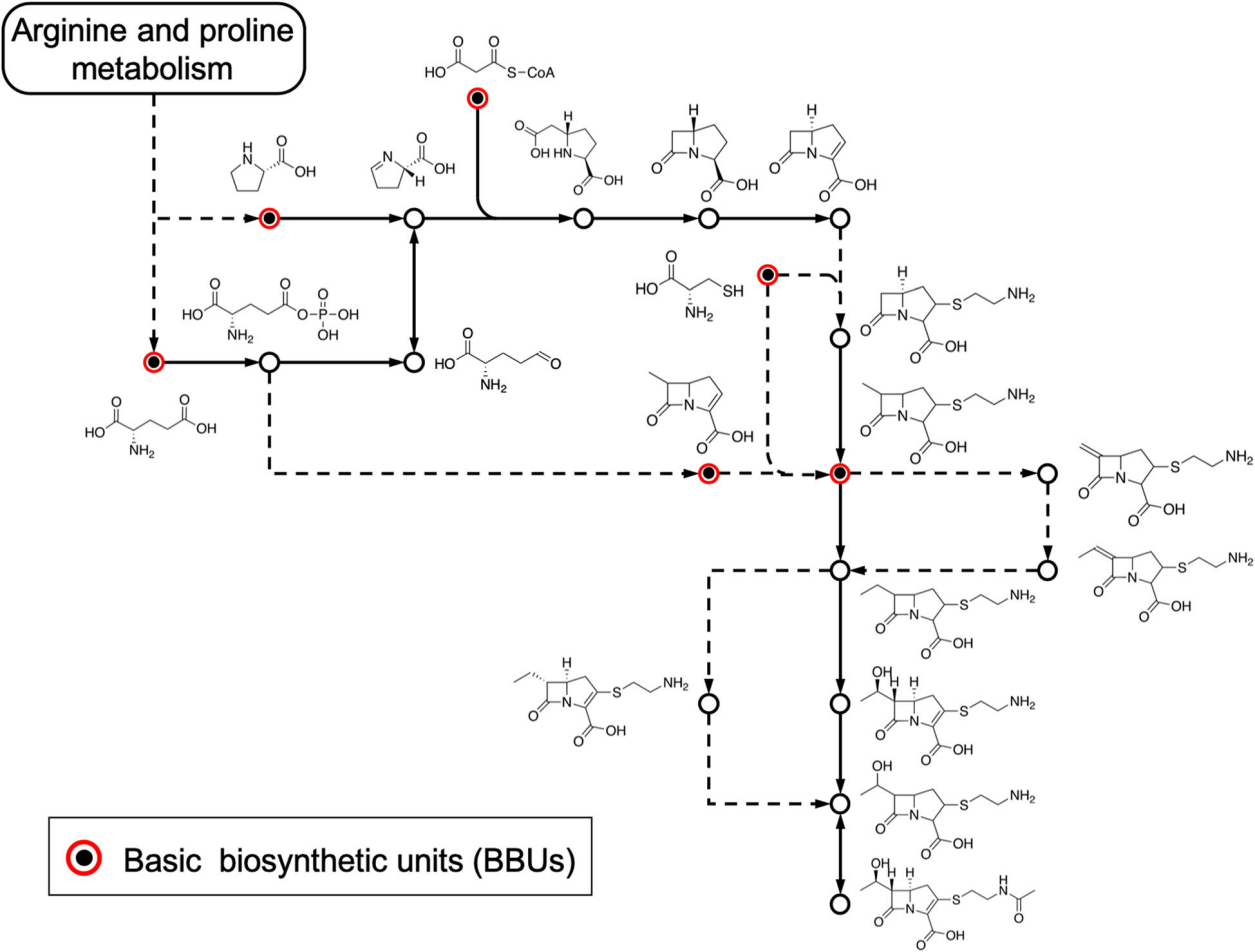
Example of a basic set of selected biosynthetic units (BBUs). BBUs were defined as important intermediates in the biosynthetic pathways. In this context, the important intermediates are the molecules in the starting point of the pathway map, the molecules at the branches of the pathway, and the molecule generated immediately after the large structural change. We followed the distinction of solid and dashed arrows as drawn in the KEGG PATHWAY database. In general, solid arrows represent the reactions for which enzymes and reactions have been well characterized, and dashed arrows represent connections to other pathway maps, or reactions that are not yet well characterized

The BBUs were defined as important intermediates in biosynthetic pathways and included the molecules in the starting point of a pathway map, the molecules at the branches of a pathway, and the molecule generated immediately after a large structural change. Examples of BBUs are shown in Fig. 1. The DBUs were identified as derivatives of BBUs by applying 14 chemical transformation rules (i.e., dehydroxylation, decarboxylation, deamination, decarbonylation, oxidative deamination, amino transfer, dentrolation, dephoshorylation, dihidrogenation/de-dihydrogenation, de-CoA, denucleotidylation, ring opening, ring closure, and hydrolysis). We obtained a set of 765 BUs from 257 BBUs and 542 DBUs. The final number was less than the sum of BBUs and DBUs because there were some common structures.

**Fig. 2:**
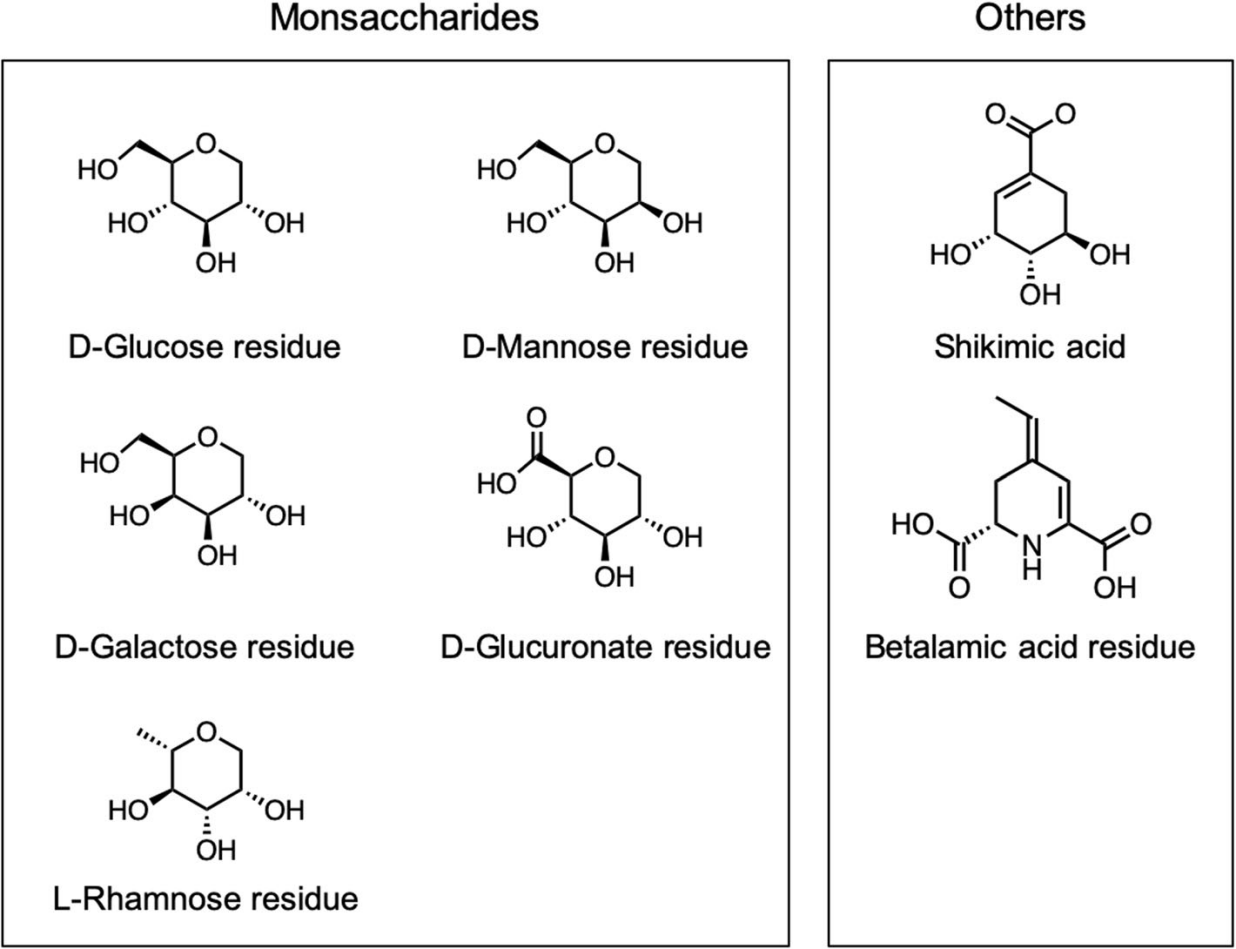
Set of preferential biosynthetic units (PBU)

Some substructures, such as the glucose residue in glucosides, remain unchanged downstream in the biosynthetic pathway. To avoid an unnecessary increase in computational costs, we defined a set of PBUs that contained five monosaccharide residues, a shikimic acid moiety, and a betalamic acid moiety (Fig. 2).

### The metabolic disassembler workflow

The workflow consists of five steps: (1) input the target molecule as a query, (2) generate a query-specific BUL, (3) generate the fragment network, (4) generate and sort the BU combination candidates, and (5) output the calculation result.

First, a user inputs the target molecule as a query. Acceptable file formats are Molfile, SMILES (Simplified Molecular Input Line Entry System) [22], InChI (International Chemical Identifier) [23], and the KEGG compound identifier [13]. The input chemical structure is converted to the Mol object of RDKit [16] and is subsequently processed using NetworkX [18].

**Fig. 3:**
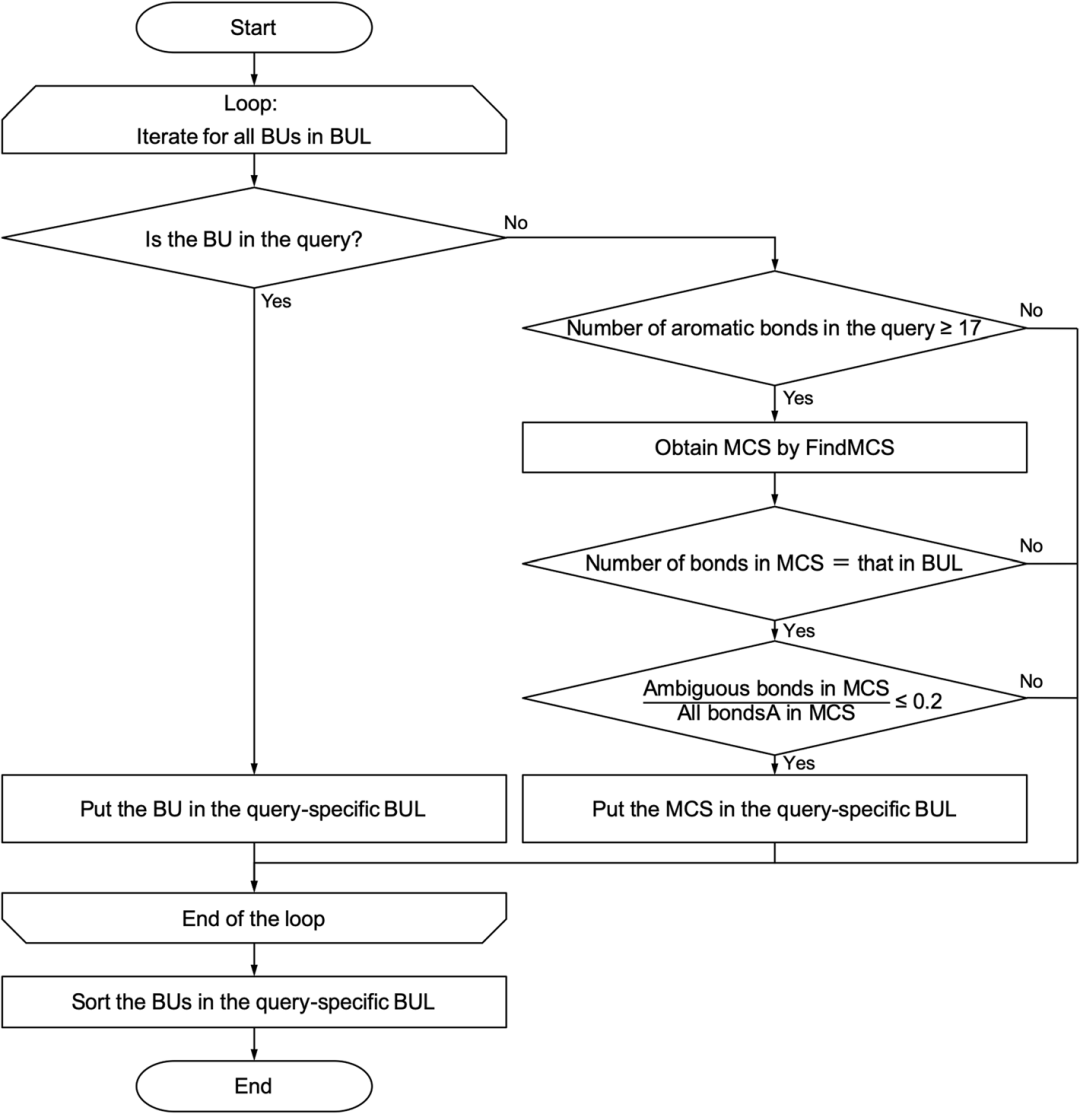
The flowchart for generating a query-specific biosynthetic unit library (BUL)

Second, each BU in the BUL is compared with the query structure. If the BU is part of the query structure, it is placed in the query-specific BUL. The aim of this process is to avoid unnecessary computation cost by removing BUs not included in the query molecule. The flowchart for generating a query-specific BUL is shown in Fig. 3. We used the HasSubstructMatch method in the RDKit library with the following option:

rdkit.Chem.HasSubstructMatch((Mol)self, (Mol)query, useChirality = True).

**Fig. 4:**
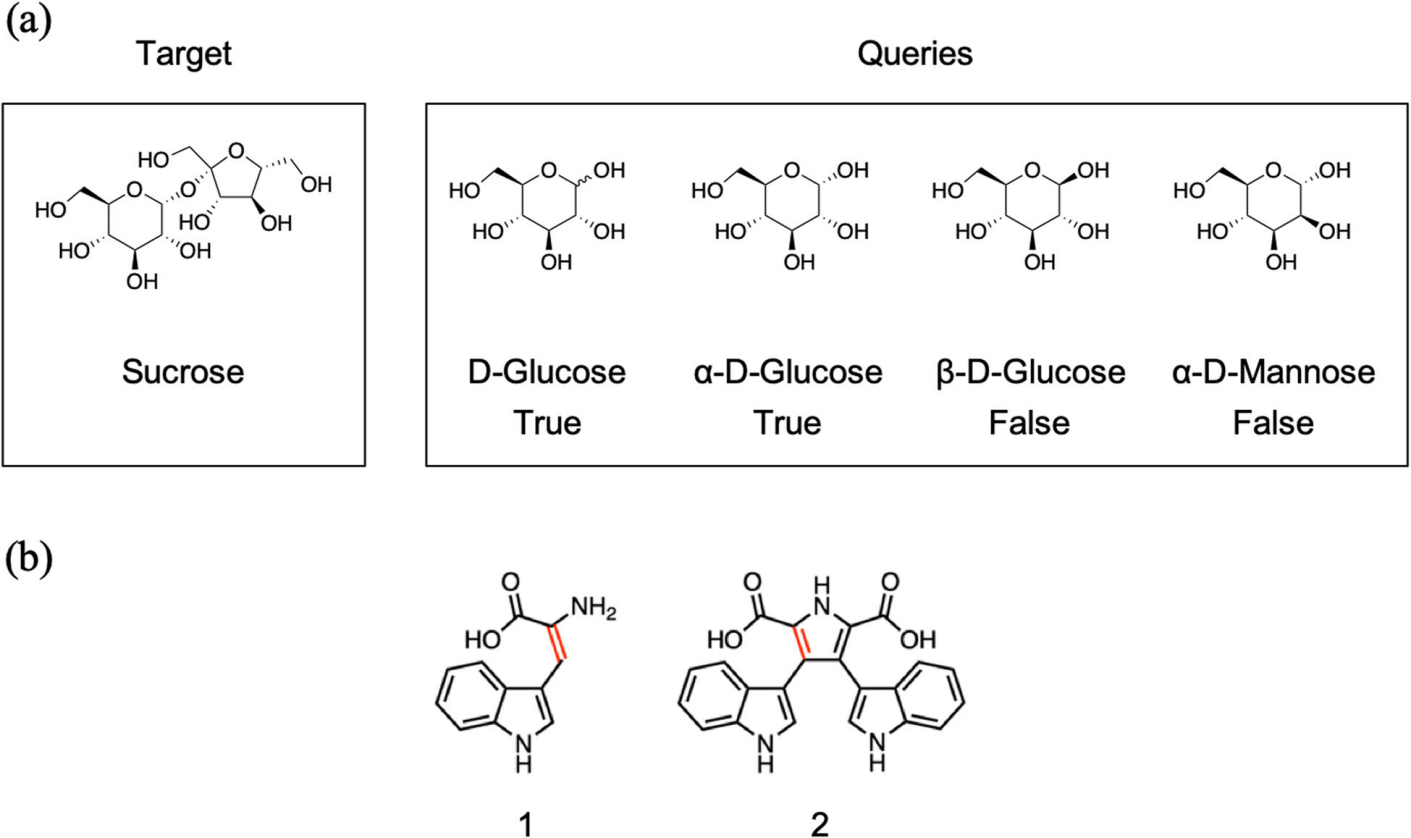
Distinguishing stereoisomers and double and aromatic bonds. (**a**) Example of distinguishing stereoisomers by RDKit.D-glucose with unclear stereoisomerism at 1-OH and α-D-glucose were regarded as being included in the target structure, whereas β-D-Glucose and α-D-Mannose were not. (**b**) Example of distinguishing double and aromatic bonds. Structure 1 was not regarded as being included in Structure 2 when using the HasSubstructMatch method in RDKit, because it distinguishes double and aromatic bonds (in red)

This method returns *True* if the query contains the given substructure, and returns *False* if not. Stereoisomers are distinguished, as shown in the example in Fig. 4a. Aromatic bonds are strictly distinguished from other conjugated double bonds, as shown in the example in Fig. 4b. This is an advantage in many cases; however, it is not useful in this study because aromatization and de-aromatization reactions are observed more frequently in biosynthetic reactions than in organic synthesis. To rescue such cases, we applied some empirical rules as follows. If the query molecule contains ≥17 aromatic bonds, the Metabolic Disassembler uses the FindMCS method [16] and rescues the falsely judged substructures.

rdkit.Chem.rdFMCS.FindMCS(mols, bondCompare = rdkit.Chem.rdFMCS.BondCompare.CompareAny).

The FindMCS method finds the maximum common substructure (MCS) in two or more molecules. Using the options as described above, bond orders such as aromatic and double bonds are ignored, thereby enabling an ambiguous MCS search. However, allowing too many ambiguous bonds produces an excessive number of candidate units. Therefore, the Metabolic Disassembler rescues a substructure only if the ratio of the number of ambiguous bonds to conjugated double bonds in the resulting MCS is ≤0.2. After collecting the units for the query-specific BUL, the units are sorted in decreasing order of the number of the atoms they contain.

**Fig. 5:**
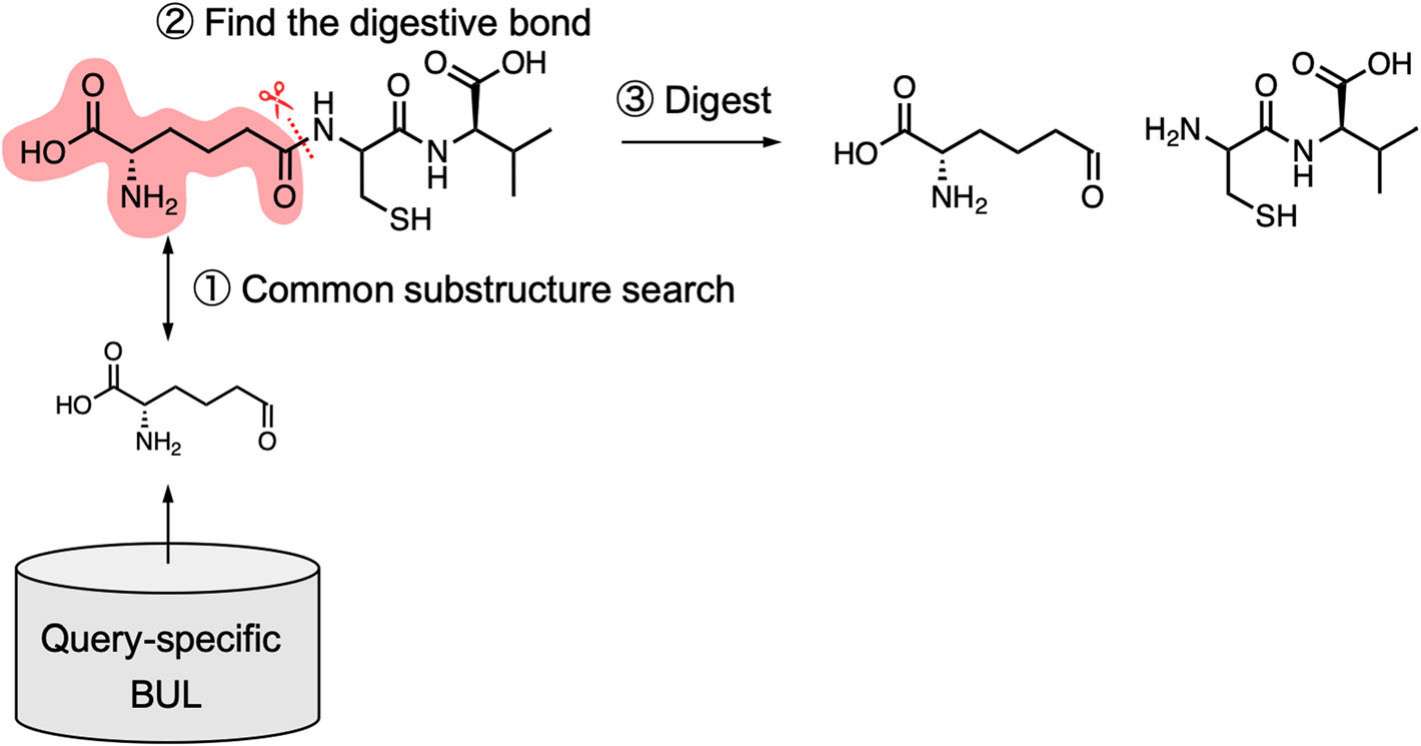
Example of the fragmentation process. One of the BUs is used in a substructure search against the target molecule to identify the bond to digest. Splitting the identified bond yields fragmentation

Third, the query molecule is divided repeatedly until every fragment matches a BU in the query-specific BUL, and a fragment network is generated to represent the relationship between the query molecule and the obtained fragments (Fig. 5). The fragment network is traversed to find the optimal combination of BUs. The MCS result is used to identify the bond that needs to be digested for fragmentation. The MCS for this process is:

rdkit.Chem.GetSubstructMatch((Mol)self, (Mol)query, useChirality = True).

**Fig. 6:**
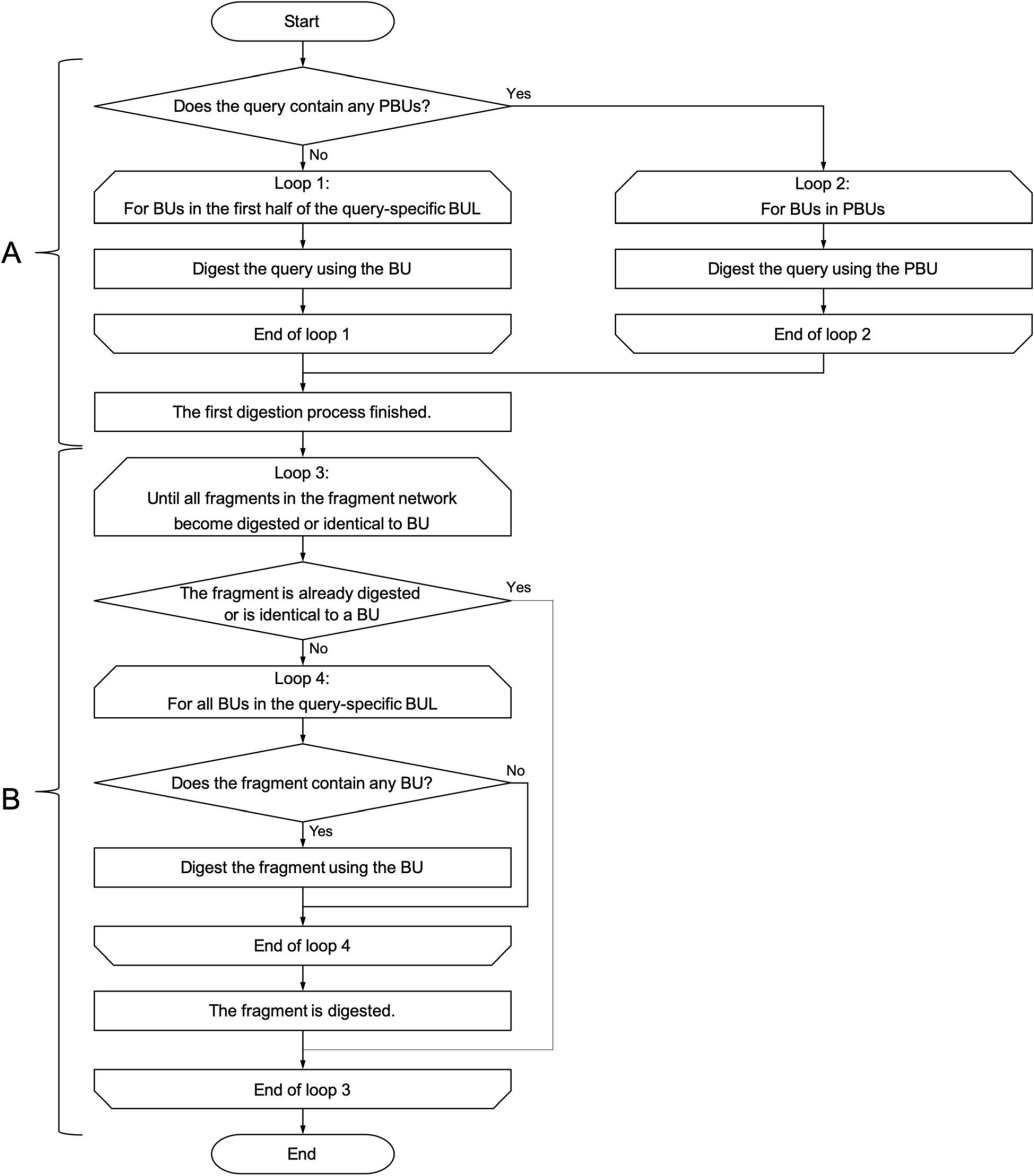
The flowchart for obtaining the fragment network

The flowchart used to obtain the fragment network has two stages (Fig. 6). In the first stage (“A” in Fig. 6), the first fragmentation is carried out by MCS against all the query-specific BUs or PBUs to find the digested bond. The second stage (“B” in Fig. 6) consists of a nested loop, which repeats digestion until every fragment matches a BU. To reduce the number of candidate BU combinations, the maximum number of selected BUs is decided by eq. (1), where N is the total number of BUs in the query-specific BUL.

n = ⌈ N/2 ⌉ (1).

**Fig. 7:**
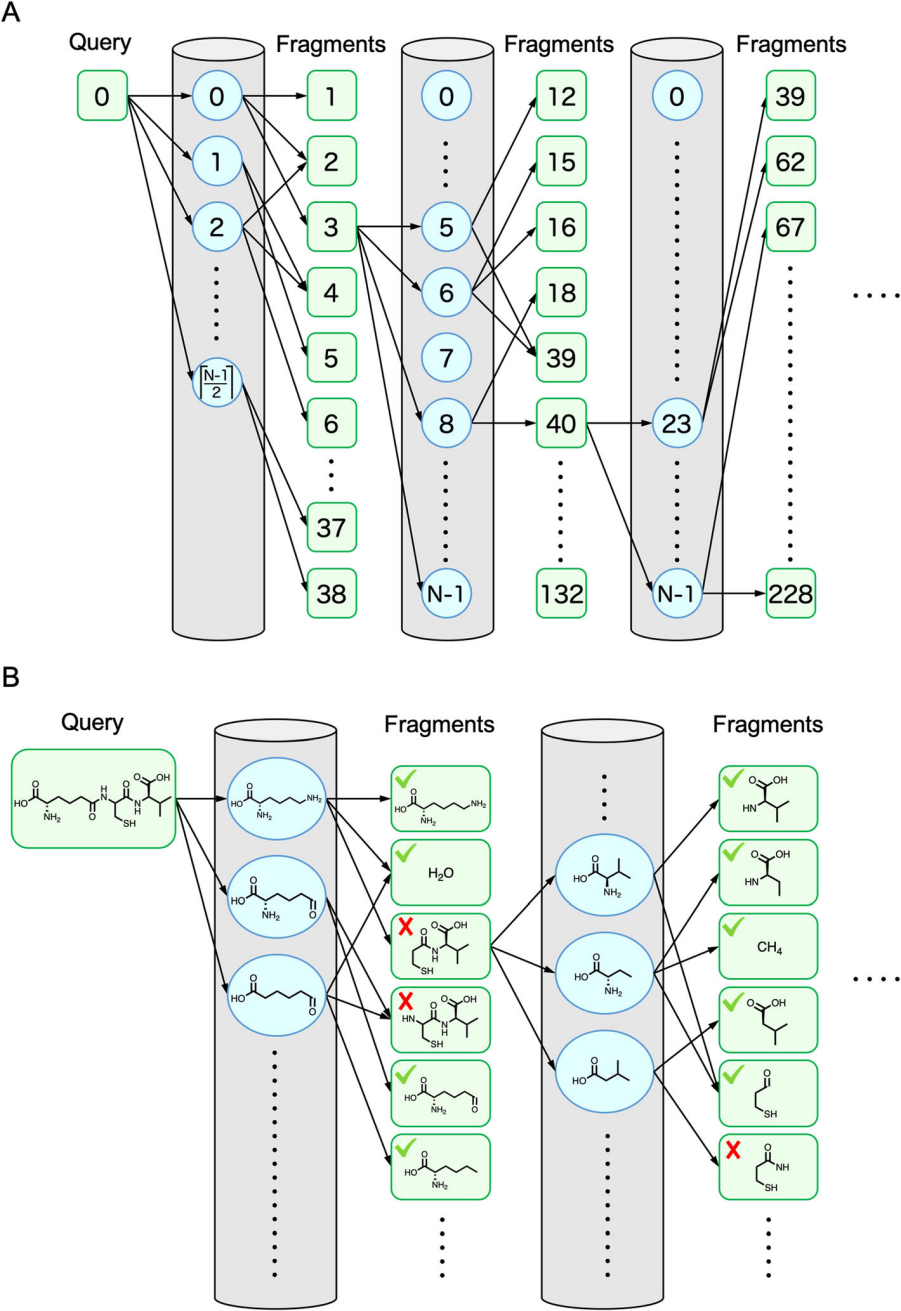
Example fragment network. (Top) Schematic diagram where the same molecules or fragments are given the same IDs. (Bottom) The same diagram where the detailed chemical structures are described

**Fig. 8:**
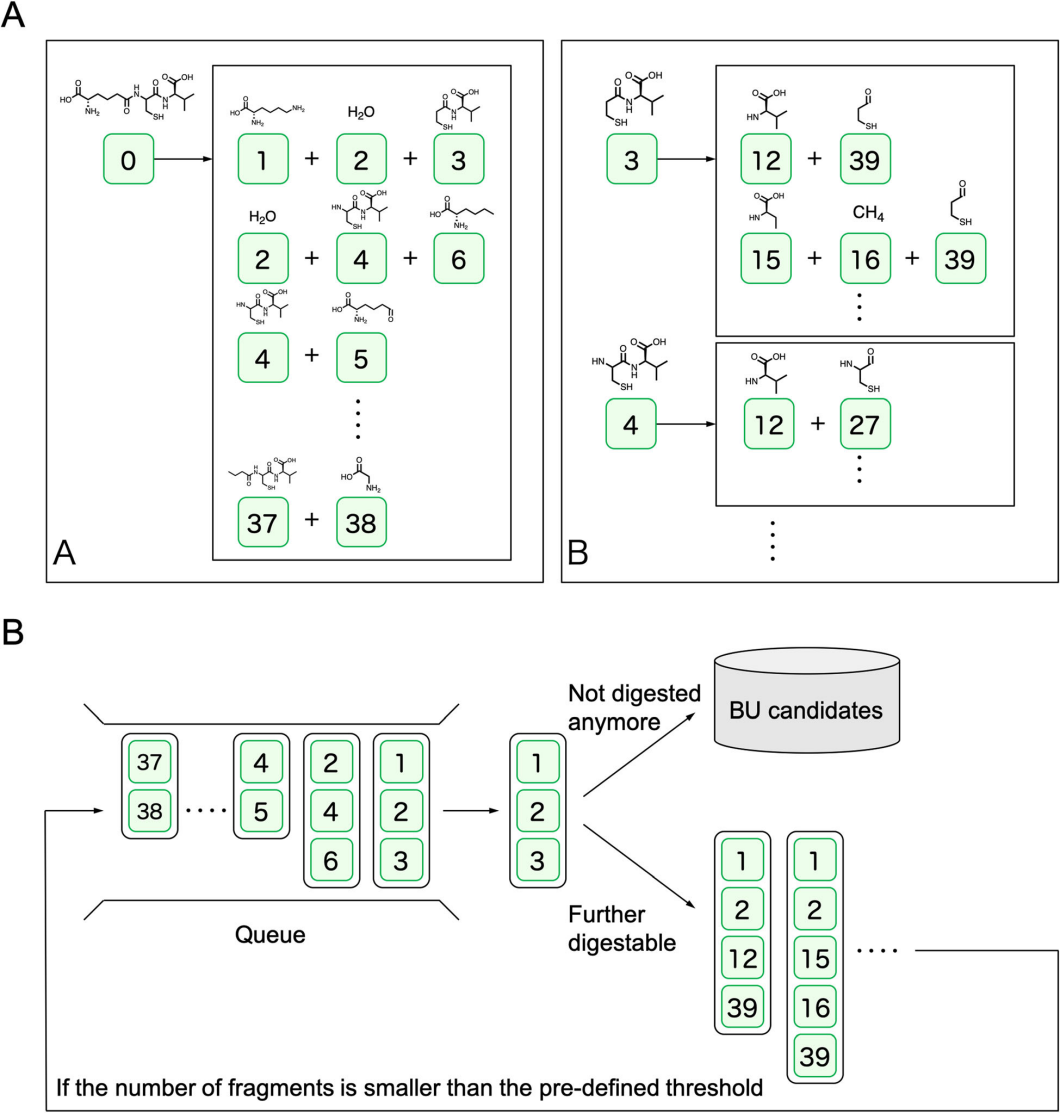
Relationship of parent and child fragments and an example of the queue structure. (**a**) Example of the parent-child relationship from the fragment network. Panel A shows the initial fragmentation; panel B shows the further fragmentation. (**b**) Queue-based procedure to obtain BU candidates

An example of a fragment network produced by the above process is shown in Fig. 7. The fragment network represents the relationship between parent (i.e., before the split) and child (i.e., after the split) fragments. An example of this relationship is shown in Fig. 8a.

Fourth, using the parent-child relationship, a parent fragment can be replaced by child fragments, and this process can be iterated using the queue data structure (Fig. 8b). The fragment combination retrieved from the queue is checked to find whether it is identical to any one of the BUs in the query-specific BUL. If it is not, the replaced combination is again inserted into the queue structure as long as the number of fragments does not exceed the upper limit, which is determined in advance by the number of atoms included in the query molecule, as defined by eq. (2).

limit_init_ = ⌈ N/k ⌉ (2).

where, k = {1 if *N* ≤ 4; 3 if 5 ≤ *N* ≤ 24; 4 if 25 ≤ *N* ≤ 53; 6 if 54 ≤ N}.

If every fragment is identical to one of the BUs in the query-specific BUL, the combination is added to the candidate list. The obtained candidate combinations are sorted in descending order of the size of the largest fragment as the first priority, and ascending order of the number of fragments as the second priority. An example of a sorted candidate list is shown in Table 1.

**Table 1:**
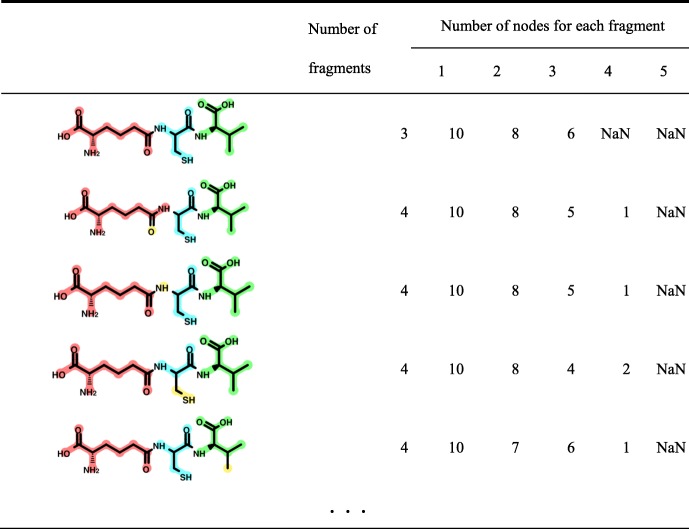
Example of the sorted biosynthetic unit (BU) candidate list. The red dotted lines indicate the bond to be digested. NaN means there are no corresponding substructures or fragments. In this example, the top candidate is the best because it contains the largest fragment, and the number of the fragment is also the largest

The red dotted lines indicate the bond to be digested. NaN means there are no corresponding substructures or fragments. In this example, the top candidate is the best because it contains the largest fragment, and the number of the fragment is also the largest

**Fig. 9:**
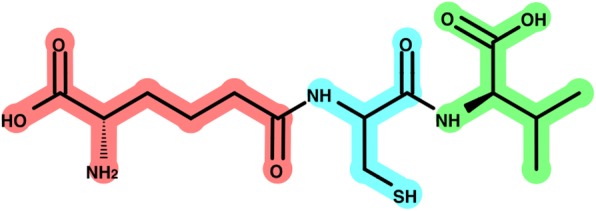
Color-coded output representing the best fragmentation

Finally, to easily understand the optimized BU combination, the Metabolic Disassembler outputs a colored image describing the fragmentation, as shown in the example in Fig. 9. Each of the split fragments is linked to the starting material.

### Comparison of the performance with the system that is not knowledge-based

**Fig. 10:**
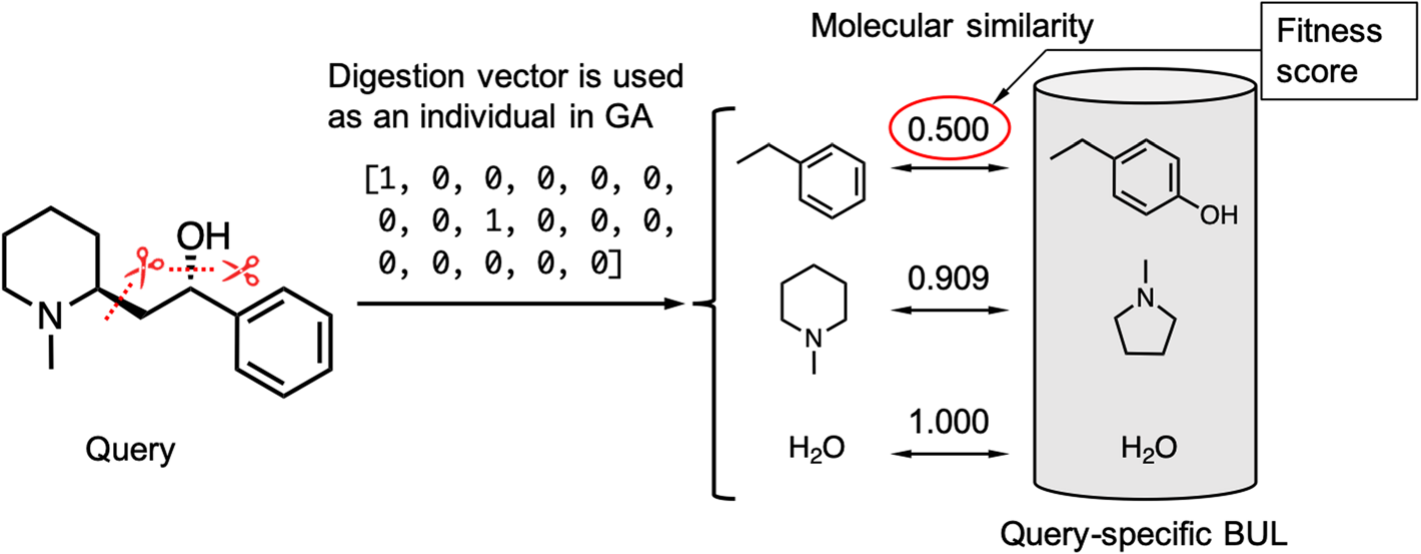
Baseline workflow using the genetic algorithm. An individual represents the combination of digested bonds, and the fitness function was defined as the minimum similarity between the fragments and the BUs in the query-specific BUL

Since none of previous studies aimed to find starting materials of secondary metabolites directly, it is difficult to show the performance of our proposed workflow. Here, the performance of our proposed workflow, which is a knowledge-based system, was compared with that of a baseline approach using a genetic algorithm, which is not knowledge-based. We used the Python library named DEAP [24] (version 1.1.2) to implement the genetic algorithm. In the genetic algorithm, the disassembly of the query molecule into BUs was solved as an optimization problem of the digested bonds in the query molecule (Fig. 10), as explained below.

First, N individuals are generated randomly as the first generation, where an individual is represented as a binary (0 or 1) vector that represents whether or not the corresponding bond should be digested. The fitness function decides whether an individual survives, and those that survive are subjected to any of three operations, namely the crossover of two individuals, mutation of an individual, or simply the copy of an individual. The generated N individuals are referred to as the next generation. This process is repeated until the predetermined ending condition is reached, and the individuals with highest fitness at the final generation are regarded as the best solution.

In this study, we set the population (the number of individuals in a generation) to 300, the crossover probability to 80%, and the mutation probability to 0.5%. The fitness function was defined as the minimum similarity between the fragments and the BUs in the query-specific BUL. We used the Tanimoto coefficient of the Morgan fingerprint (radius 2, 1024 bit) [25] as the similarity measure.

## Results

### Performance evaluation

A summary of the ratio of correct disassembly for the respective pathway maps is shown in Table 2. Note that the Metabolic Disassembler inputs only the chemical structure of the query molecule and does not require any pathway information. We used the pathway maps only for the performance evaluation. We found that 903 of the 943 molecules (approximately 96%) were disassembled correctly. Detailed results are presented in Additional file 1.

**Table 2:** Accuracy of the proposed Metabolic Disassembler workflow for each metabolic map

Map number	Name of the pathway map	Correctness for the first candidate	Correctness for the other candidates	Incorrect	Number of molecules	Accuracy
00231	Puromycin biosynthesis	10	0	0	10	1.000
00232	Caffeine metabolism	14	0	0	14	1.000
00254	Aflatoxin biosynthesis	17	0	4	21	0.810
00261	Monobactam biosynthesis	18	6	0	24	1.000
00311	Penicillin and cephalosporin biosynthesis	12	0	0	12	1.000
00331	Clavulanic acid biosynthesis	6	2	0	8	1.000
00332	Carbapenem biosynthesis	22	0	1	23	0.957
00333	Prodigiosin biosynthesis	1	0	10	11	0.091
00401	Novobiocin biosynthesis	17	0	1	18	0.944
00402	Benzoxazinoid biosynthesis	6	0	0	6	1.000
00403	Indole diterpene alkaloid biosynthesis	30	0	0	30	1.000
00404	Staurosporine biosynthesis	32	0	0	32	1.000
00405	Phenazine biosynthesis	17	5	3	25	0.880
00521	Streptomycin biosynthesis	15	0	0	15	1.000
00524	Neomycin, kanamycin and gentamicin biosynthesis	67	0	2	69	0.971
00525	Acarbose and validamycin biosynthesis	29	0	0	29	1.000
00901	Indole alkaloid biosynthesis	54	1	1	56	0.982
00940	Phenylpropanoid biosynthesis	51	0	4	55	0.927
00941	Flavonoid biosynthesis	63	0	0	63	1.000
00942	Anthocyanin biosynthesis	63	0	0	63	1.000
00943	Isoflavonoid biosynthesis	55	2	2	59	0.966
00944	Flavone and flavonol biosynthesis	49	0	0	49	1.000
00945	Stilbenoid, diarylheptanoid and gingerol biosynthesis	21	2	0	23	1.000
00950	Isoquinoline alkaloid biosynthesis	98	0	1	99	0.990
00960	Tropane, piperidine and pyridine alkaloid biosynthesis	44	3	11	58	0.810
00965	Betalain biosynthesis	20	0	0	20	1.000
00966	Glucosinolate biosynthesis	48	5	0	53	1.000
01058	Acridone alkaloid biosynthesis	14	1	0	15	1.000
	Total (without redundancy)	876	27	40	943	0.958

### Computational time

**Fig. 11:**
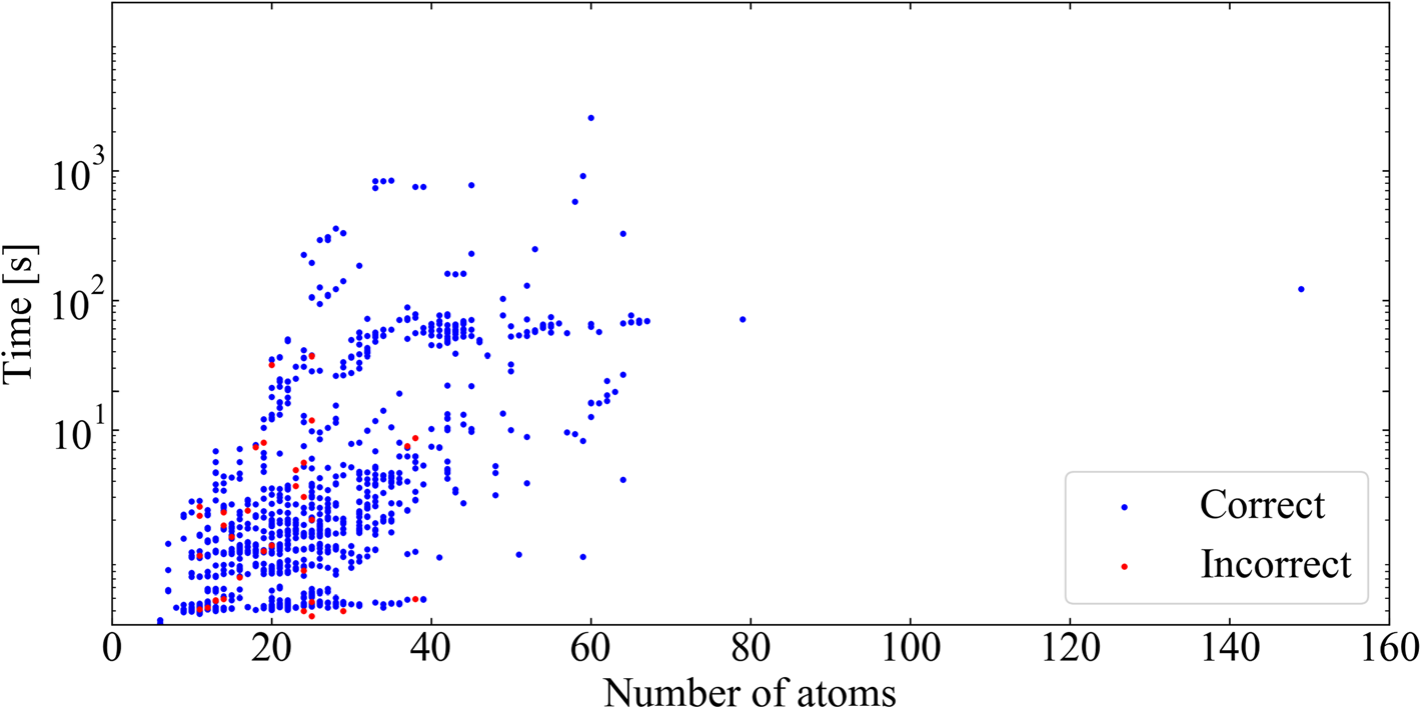
Plot of computational time for each molecule. The computational time is given using a log scale

We measured the computational time for the Metabolic Disassembler with the 943 molecules, executed in the computing environment described in Methods. We performed the calculation five times for each molecule and the obtained average values were recorded (Table 3). The plot of the computational time for each molecule is shown in Fig. 11. One of the molecules needed more than one day for the calculation, so it was excluded from the result. Therefore, the total number of molecules disassembled was 942. Among them, 855 molecules (approximately 91%) were each disassembled within one minute, indicating the processing speed was sufficiently practical in the current implementation. Sixteen molecules took more than 5 min to disassemble (Table 4). One of the causes of the increased execution time came from the application of the FindMCS method in RDKit.

**Table 3:** Distribution of computational times for correct and incorrect disassembly

	Runtime (s)				
	Average	Median	SD	Max	Min
Correct answers (*n* = 903)	27.5	2.0	121.0	2566.0	0.3
Incorrect answers (*n* = 39)	4.0	1.2	7.8	37.1	0.1
All (*n* = 942)	26.5	2.0	118.6	2566.0	0.1

**Table 4:**
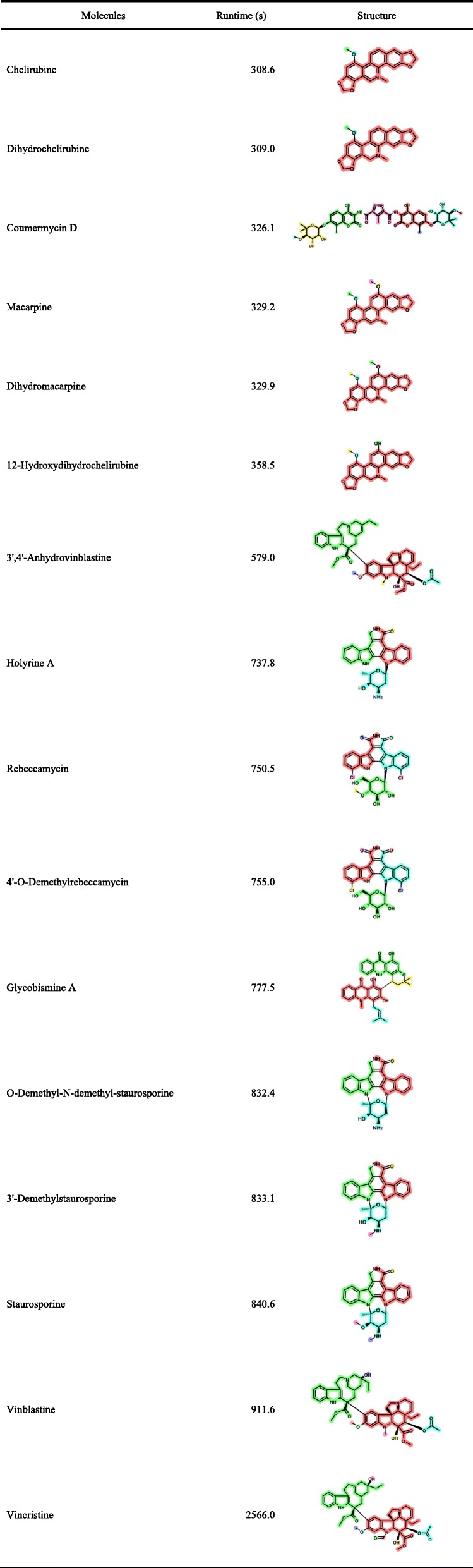
The molecules that needed more than five minutes of computational time for disassembly

### Comparison between the metabolic disassembler and the baseline workflow using a genetic algorithm

**Fig. 12:**
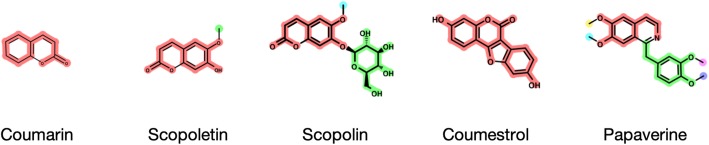
The molecules that were correctly disassembled by the baseline workflow but not by the proposed workflow

For the fair comparison to our proposed workflow, we measured the computational time of the baseline workflow and decided the number of generations to 200, so that it takes approximately similar range of computational time. The average and maximum runtimes were 252 and 1512 s, respectively, showing that the computational time was not different significantly from our proposed workflow. Also, in the evaluation process of the baseline workflow, we took top five most fitted individuals, and regarded as correct if one of them is correctly disassembled. Table 5 shows the comparison of our proposed workflow and the baseline workflow, and details of the numbers of correctly and incorrectly disassembled molecules using the baseline workflow are shown in Table 6. Among the molecules that were not correctly disassembled by the Metabolic Disassembler, five were correctly disassembled by the baseline workflow (Fig. 12). The baseline workflow does not depend on the distinction of aromatic bonds, which led to these successful cases. However, our proposed workflow generally performed better than the baseline workflow. The baseline workflow used the Tanimoto coefficient of Morgan fingerprint, which takes values from 0.0 (dissimilar) to 1.0 (similar). Importantly, 1.0 does not always mean identical. In addition, when the molecular similarity is low it is more difficult to interpret the obtained fragment. The proposed workflow performs exact matching, which provides easier interpretation.

**Table 5:** Comparison of the performance of the proposed Metabolic Disassembler workflow and the baseline workflows

	Correct	Incorrect	Accuracy
Proposed workflow	903	40	0.958
Baseline workflow using the genetic algorithm	490	453	0.520

**Table 6:** Accuracy of the baseline workflow for each metabolic map

Map number	Name of the pathway map	Correct	Incorrect	Number of molecules	Accuracy
00231	Puromycin biosynthesis	4	6	10	0.400
00232	Caffeine metabolism	11	3	14	0.786
00254	Aflatoxin biosynthesis	13	8	21	0.619
00261	Monobactam biosynthesis	9	15	24	0.375
00311	Penicillin and cephalosporin biosynthesis	8	4	12	0.667
00331	Clavulanic acid biosynthesis	8	0	8	1.000
00332	Carbapenem biosynthesis	13	10	23	0.565
00333	Prodigiosin biosynthesis	1	10	11	0.091
00401	Novobiocin biosynthesis	13	5	18	0.722
00402	Benzoxazinoid biosynthesis	6	0	6	1.000
00403	Indole diterpene alkaloid biosynthesis	13	17	30	0.433
00404	Staurosporine biosynthesis	11	21	32	0.344
00405	Phenazine biosynthesis	4	21	25	0.160
00521	Streptomycin biosynthesis	4	11	15	0.267
00524	Neomycin, kanamycin and gentamicin biosynthesis	39	30	69	0.565
00525	Acarbose and validamycin biosynthesis	20	9	29	0.690
00901	Indole alkaloid biosynthesis	30	26	56	0.536
00940	Phenylpropanoid biosynthesis	25	30	55	0.455
00941	Flavonoid biosynthesis	42	21	63	0.667
00942	Anthocyanin biosynthesis	32	31	63	0.508
00943	Isoflavonoid biosynthesis	32	27	59	0.542
00944	Flavone and flavonol biosynthesis	37	12	49	0.755
00945	Stilbenoid, diarylheptanoid and gingerol biosynthesis	10	13	23	0.435
00950	Isoquinoline alkaloid biosynthesis	31	68	99	0.313
00960	Tropane, piperidine and pyridine alkaloid biosynthesis	16	42	58	0.276
00965	Betalain biosynthesis	7	13	20	0.350
00966	Glucosinolate biosynthesis	47	6	53	0.887
01058	Acridone alkaloid biosynthesis	12	3	15	0.800
	Total (without redundancy)	490	453	943	0.520

## Discussion

Our proposed workflow correctly disassembled 903 molecules, but incorrectly disassembled 40 molecules. The causes of the incorrect disassembly are given in Table 7.

**Table 7:** Causes of incorrect disassembly

Map number	MCS search	Change in ring structures	Use of PBUs	Fatty acid and polyketide biosynthesis	Shortage of BUs	Others	Total
00254	0	4	0	0	0	0	4
00332	0	0	0	0	0	1	1
00333	5	0	0	5	0	0	10
00401	0	0	0	0	0	1	1
00405	3	0	0	0	0	0	3
00524	0	0	0	0	2	0	2
00901	0	0	1	0	0	0	1
00940	3	0	0	0	1	0	4
00943	2	0	0	0	0	0	2
00950	1	0	0	0	0	0	1
00960	5	0	0	0	6	0	11
Total	19	4	1	5	9	2	40

**Fig. 13:**
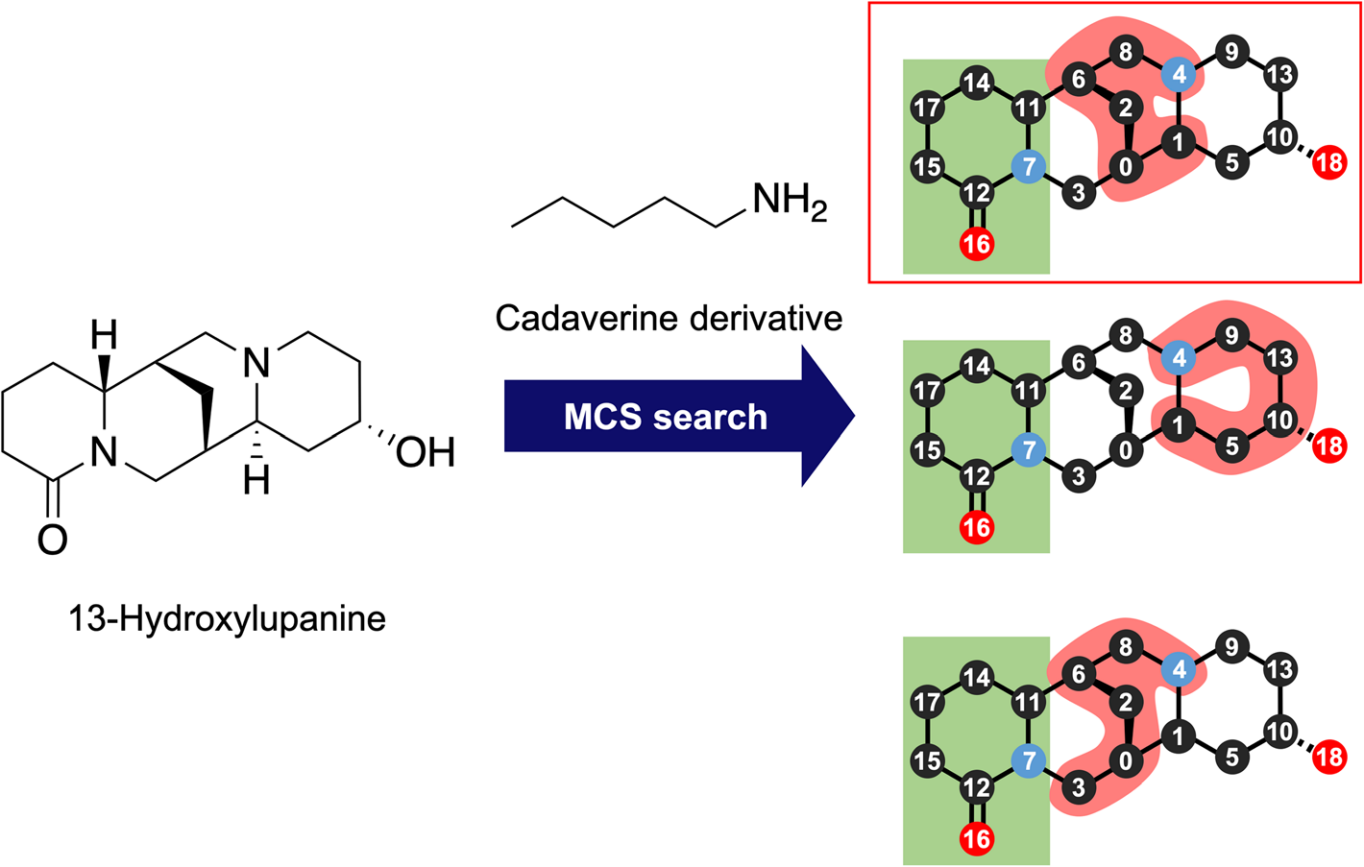
Substructure search of cadaverine against 13-hydroxylupanine.The substructures highlighted in red correspond to the query cadaverine. The substructures in green rare those already matched to other substructures. The GetSubstructMatch method of RDKit returns only the first result found, even though theoretically there are more possible results

The substructure search methods caused most of the incorrect results. The HasSubstructMatch and GetSubstructMatch methods in RDKit were used for the substructure search, and these methods distinguish aromatic bond from conjugated double bonds. Our workflow allowed ambiguous bonds in limited circumstances, making it possible to cope with the metabolic reaction to form an aromatic ring by ring closure. However, three molecules, coumestrol (C10205), phenazine-1,6-dicarboxylic acid (C12119), and papaverine (C06533), were incorrectly disassembled. We decided not to apply this rule if the number of aromatic bonds in the query molecule was ≤16 and because of this, six molecules, anhydroglycinol (C10200), 2-heptyl-4-quinolone (C20643), 2-heptyl-3-hydroxy-4-quinolone (C20643), coumarin (C05851), scopoletin (C01752), and scopolin (C01527), were incorrectly disassembled. Among the nine incorrectly disassembled molecules, coumarin, scopoletin, scopolin, coumestrol, and papaverine were correctly disassembled by the baseline approach, as explained above. However, the other four molecules were incorrectly disassembled by both workflows. We used the FindMCS method in RDKit to allow ambiguous matching, but this method easily leads to an increase in the calculation time and a decrease in interpretability. Additionally, the five molecules in map00960, slaframine (C06185), 13-(2-methylcrotonoyl)oxylupanine (C04170), 13-hydroxylupanine (C02621), pseudopelletierine (C10865), and calystegin A3 (C10850), were incorrectly disassembled despite of the presence of relevant BUs. This was caused by the GetSubstructMatch method in RDKit, which returns only the first found substructure even if theoretically there are more substructures, as shown in the example in Fig. 13. This problem could be solved by using GetSubstructMatches in RDKit, which returns all possible combinations, instead of GetSubstructMatch. However, this method easily causes an increase in the calculation time and, thus, it is not efficient enough to be applied in a simple way. Therefore, the future improvement of these problems would include the development of a more suitable method for the substructure search.

Some of the other unsuccessful disassembles were caused by rearrangements of the ring structures. For example, the structures of the fused rings in aflatoxin B1 (C06800), B2 (C16753), G1 (C16755), and G2 (C16754) were different from those of the respective precursors, which caused the incorrect disassembly. This problem could be remedied by adding more substructures to the BUL; however, this is not realistic.

**Fig. 14:**
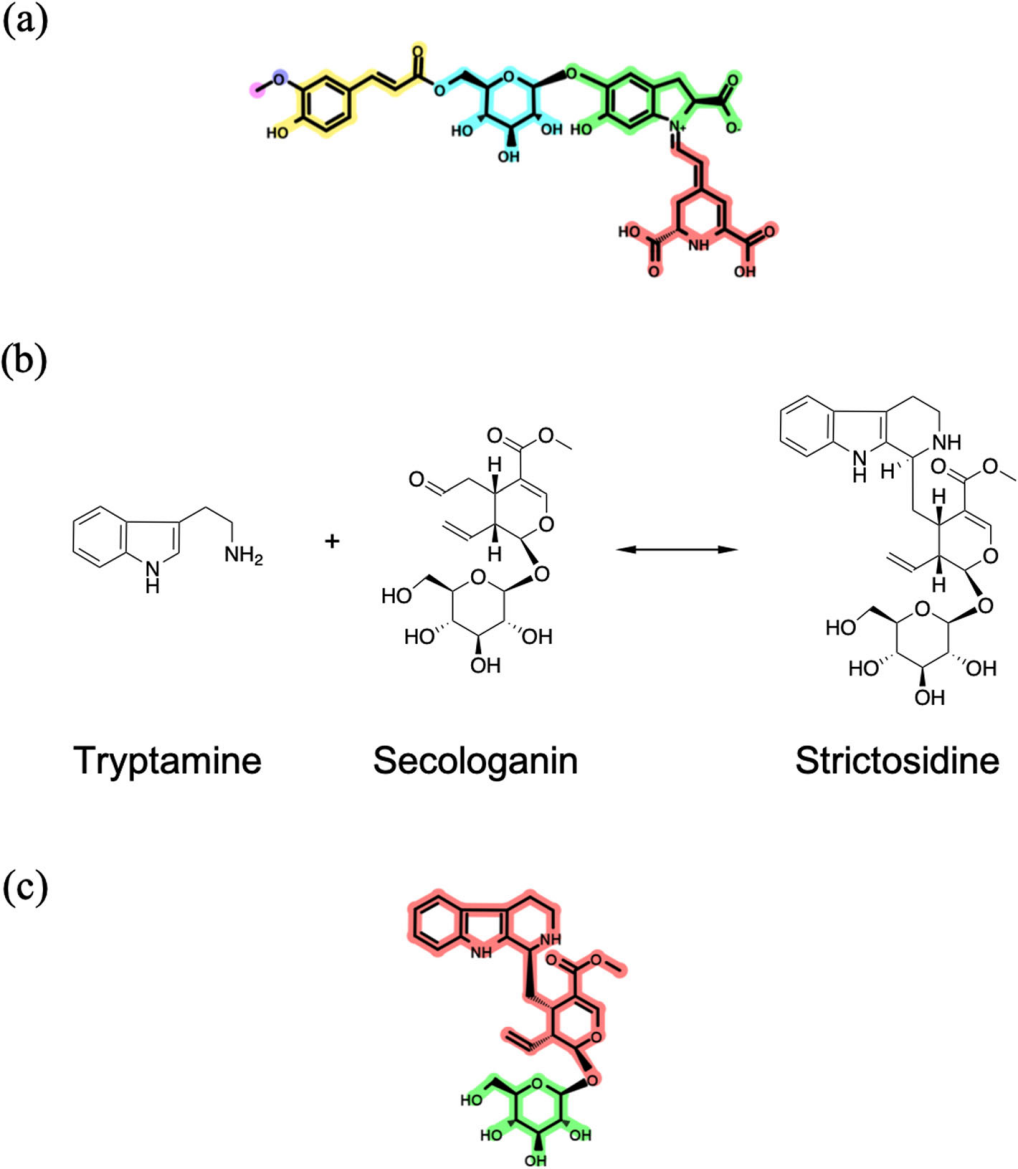
Effects of preferential biosynthetic units on disassembly by the Metabolic Disassembler. (**a**) Disassembly of lampranthin II. (**b**) Biosynthesis of strictosidine. (**c**) Incorrect disassembly of strictosidine

We introduced PBUs with the aim of shortening the calculation time, especially for molecules that contain sugar residues. We also compared the algorithms that did and did not use PBUs, taking lampranthin II (C08552), which has a betalamic acid (C08538) moiety and saccharide residues, as an example (Fig. 14a). The computational time was measured five times both before and after the introduction of the PBUs. The computational times before and after the introduction of the PBUs were 208.58 ± 2.46 s and 4.38 ± 0.11 s, respectively; therefore, we concluded that introducing the PBUs significantly improved the computational speed. We found a case where introducing the PBUs led to an incorrect disassembly (Fig. 14b, c); however, this is the only incorrect case that we found, implying this was not a major problem.

In this study, we did not focus on terpenoids and polyketides, which were mainly in the metabolism map 1.9 class. The appropriate BUs of terpenoids and polyketides would be acetyl-CoA and so on, however, adding these BUs could not solve the problem. In this and other studies, manual curations are important for good performance. BBUs and DBUs were manually selected in order to appropriately disassemble molecules, and PBUs were selected in order to shorten the unnecessary computational time. Enhancement of the BUL could improve the coverage but may increase the incorrect substructure matches. We already examined our manual curation, and the current BUs are the best at this moment. When it becomes apparent that some other secondary metabolites originate from other pathways, the enhancement of BUs may work well.

In fact, the 1.10 class also contains some terpenoids and polyketides and our proposed workflow produced incorrect disassembles for them. The four molecules in map000333, 2-methyl-3-n-amyl-dihydropyrrole (C21571), 2-methyl-3-n-amyl-pyrrole (C21572), 4-keto-2-undecylpyrroline (C21573), and 2-undecylpyrrole (C21574), are generated by amination and cyclization of fatty acids, but do not originate from L-proline as incorrectly indicated by our proposed workflow. Similarly, for the fatty acid dodecanoic acid (C02679) in map000333, the desired combination is the six C_2_ units that originate from malonyl-CoA (C00083). However, this molecule was excluded from the calculation because the number of fragments exceeded the upper limit.

The BUL could not support cases where the number of atoms decreased in the BU or when a double bond was generated in a BU by cyclization and subsequent oxidation. For example, to cope with the C_7_ units derived from octanoyl-CoA (C01944), it is necessary to prepare 19 BUs in which one of the six C–C bonds becomes a double bond. There are 33 combinations for the C_8_ units. Therefore, preparing for other C_n_ units produces an enormous number of BUs and slows the processing speed. In addition, if many straight-chain alkyl groups are registered in the BUL, molecules that did not originate from acetate-malonic acid pathway are adversely affected. Therefore, we concluded that the BUL and our proposed workflow should not be applied to the biosynthesis of terpenoids and polyketides.

**Fig. 15:**
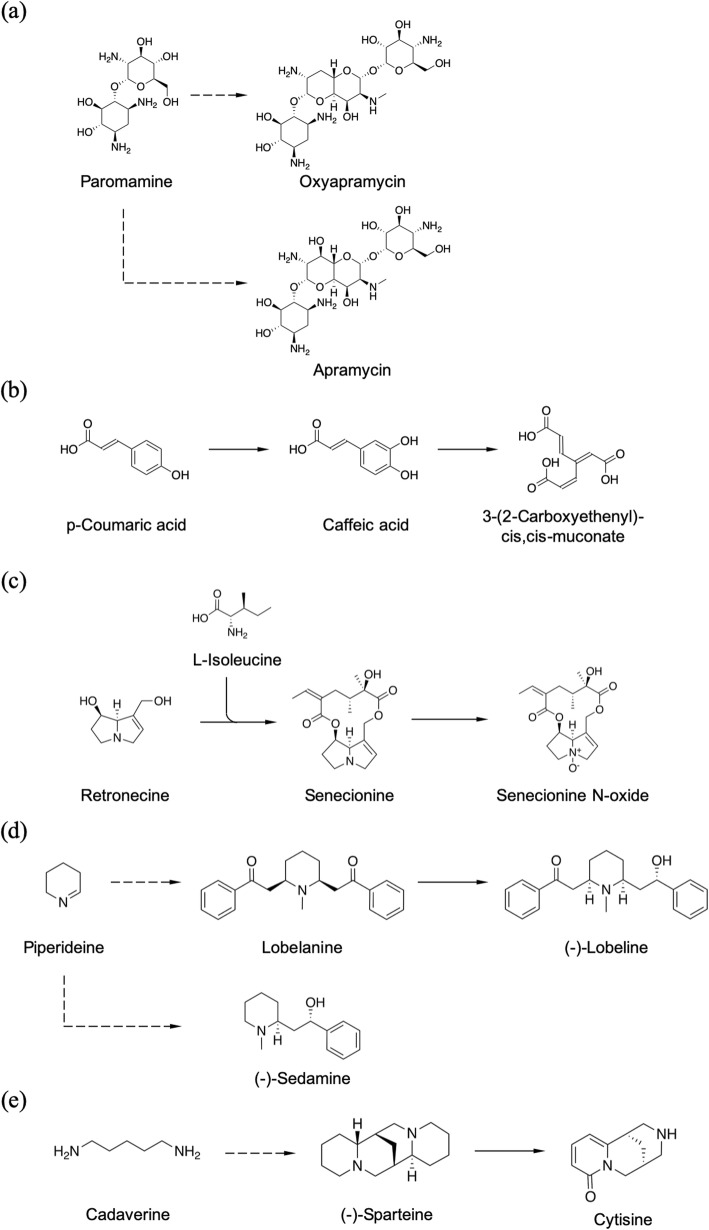
Biosynthesis of the molecules that were not covered the BUL. (**a**) Apramycin and oxyapramycin. (**b**) 3-(2-carboxyethenyl)-cis,cis-muconate. (**c**) Senecionine. (**d**) Lobelanine and (−)-lobeline and (−)-sedamine. (**e**) Cytisine

Despite the efforts to enhance the BUL to cover a wide range of natural products, there were still nine molecules that were not covered. Apramycin (C01555) and oxyapramycin (C17997) in map00524 are synthesized via paromamine (C01743; Fig. 15a). These two molecules look like dimers but not exactly, and their biosynthetic pathways are not yet apparent. They contain substructures other than the paromamine (C01743) residue and their origins are unclear, and could not be dealt with by the proposed workflow. 3-(2-Carboxyethenyl)-cis,cis-muconate (C04366) in map00940 is synthetized by oxidative cleavage of the benzene ring of caffeic acid (C01197; Fig. 15b). This molecule can be dealt with by preparing a relevant BU, but such cleavage was observed only in this molecule. Therefore, we decided we should not prepare such a BU considering its low general use. Senecionine (C06176) and senecionine N-oxide (C15612) in map00960 are synthesized by condensation of retronecine (C06177) and two L-isoleucine (C00407; Fig. 15c). A carbon atom from each L-isoleucine unit is eliminated during the biosynthesis, but the reaction mechanism is not clear. Lobelanine (C10157), (−)-lobeline (C07475), and (−)-sedamine (C10171) originate from piperideine (C06181), but their biosynthetic mechanisms are not clear (Fig. 15d). Cytisine (C10763) is biosynthesized by conjugating cadaverine (Fig. 15e); however, four carbon atoms are eliminated during the biosynthesis, but its complete biosynthetic mechanism has not been revealed. Therefore, appropriate BUs could not be prepared for these molecules.

Although enhancement of the BUL could be a solution, its expansion may increase the number of incorrect substructure matches as well as the calculation time. A sufficient number of BUs is necessary for successful disassembly, therefore, it is necessary to strengthen the BUL while considering its tradeoff.

### Conclusions

The Metabolic Disassembler disassembles target chemical structures into relevant biosynthetic units that correspond to their starting materials, which is the first step in predicting the biosynthetic pathways of natural products. The users can use the Python program as well as the BUL so that the users do not have to reproduce this knowledge-based system. The Metabolic Disassembler will also help to identify the chemical bonds generated during the biosynthetic pathway, thereby providing valuable information for predicting the biosynthetic pathway of natural products.

## Supplementary information

**Additional file 1.** Summary of disassembly calculation for each map. Detailed explanation of disassembly calculation for each pathway map.

## Data Availability

The Metabolic Disassembler is freely available at https://github.com/the-metabolic-disassembler/metadisassembler.

## Abbreviations

BBU: basic BU
BU: biosynthetic unit
BUL: biosynthetic unit library
DBU: derivative BU
PBU: preferential BU
