# Supplementary material for: Metabolic disassembler for understanding and predicting the biosynthetic units of natural products
Source: BMC Bioinformatics. 2019 Dec 23;20:728. doi: 10.1186/s12859-019-3183-9 (PMC6929434; doi:10.1186/s12859-019-3183-9)
Supplement: Supplementary file 1 — Additional file 1. Summary of disassembly calculation for each map. Detailed explanation of disassembly calculation for each pathway map. [file 12859_2019_3183_MOESM1_ESM.docx]

Supplementary Material

Details of the prediction of the metabolic disassembler

**map00231 Puromycin biosynthesis**

Puromycin (C01610) is an aminoacyl nucleoside antibiotic produced by *Streptomyces alboniger*, and it is known to block protein synthesis by inhibiting the elongation of the peptide chain. Puromycin is synthesized by forming a peptide-like bond between L-tyrosine (C00082) and 3'-amino-3'-deoxy-AMP (C07026), which is originated from ATP (C00002), followed by N-acetylation, N-dimethylation, O-methylation, and the N-deacetylation [1]. We confirmed that the 10 molecules in map00231, including puromycin (C01610, Fig. 1), were successfully disassembled into units corresponding to their biosynthetic mechanism (the entire prediction result is described in Fig. S-1).

**map00232 Caffeine metabolism**

The basic skeleton of the caffeine metabolism has a purine ring composed of the six-membered ring and the five-membered ring of carbon and nitrogen atoms (Fig 2). This basic skeleton is introduced not only from caffeine (C07481), but also from xanthosine (C01762). Therefore, by preparing a derivative of this basic skeleton as a DBU, the correct prediction was made possible for almost all compounds on this map. The remaining molecules are synthesized by cleaving the purine ring of paraxanthine (C13747). We defined the molecule that is generated after the cleavage as a BBU, which enabled the disassembly of the remaining molecules correctly (the entire prediction result is described in Fig. S-2).

**map00254 Aflatoxin biosynthesis**

Aflatoxins (*e.g*., C06800) are toxins produced by *Aspergillus*, and it has a common substructure consisting of bisfuran ring and coumarin. The first intermediate, norsolorinic acid (C20452), is synthesized from acetyl-CoA (C00024) and malonyl-CoA (C00083) by a polyketide synthase. The next intermediate, 1'-hydroxyversicolorone (C20503), is generated by the combination of cyclization and a rearrangement of the ring structure. Aflatoxins are synthesized after another rearrangement of the ring. It is difficult to apply the BUs when such rearrangement occurs. This was supposed to be the reason why four molecules were not correctly disassembled (Fig 3). Still, the other 17 molecules were correctly disassembled (the entire prediction result is described in Fig. S-3).

**map00261 Monobactam biosynthesis**

Monobactams are β-lactam antibiotics having a single β-lactam ring in the basic skeleton. Nocardicins are natural monobactams, and are synthesized via nocardicin G (C17355), which is a pentapeptide generated by condensation of L-4-hydroxyphenylglycine (C12323), L-arginine (C00062), and L-serine (C00065) [2]. The biosynthetic pathways of other natural monobactams on the map are not yet clear. Aztreonam (C06840) was the first clinically used monobactam, and was isolated as SQ 26180 (C16842) from *Chromobacterium violaceum*. Tabtoxinine-β-lactam (C20918) is induced from tabtoxin (C20917), a tobacco wildfire pathogen. Biosynthetic machinery of many molecules in this map are not yet clear, and the correctness of the disassemble results for sulfazecin (C20927), SQ 26180 (C16842) and MM 42842 (C20928) were estimated from the biosynthetic machinery of nocardicins (Fig 4). As a result, we concluded that all 24 molecules were disassembled correctly (the entire prediction result is described in Fig. S-4).

**map00311 Penicillin and cephalosporin biosynthesis**

Penicillins and cephalosporins are classified into β-lactam antibiotics. First, a non-ribosomal peptide synthetase (NRPS) synthesize an intermediate named ACV (C05556) from three amino acids, L-2-aminoadipate (C00956), L-cysteine (C00097), and L-valine (C00183) [3]. Penam skeleton (Fig. 5) is a basic skeleton of penicillins, and is made from L-cysteine, and L-valine. We confirmed that they could be correctly disassembled as shown in Fig 6. Cephem skeleton (Fig. 5) is a basic skeleton of cephalosporin antibiotics, and is made by the rearrangement of the penam skeleton to form a hetero six-membered ring. Compounds where the penam skeleton changes to the cephem skeleton were recorded as a BBU. As a result, all the cephalosporin antibiotics on the map could be correctly disassembled (the entire prediction result is described in Fig. S-5).

**map00331 Clavulanic acid biosynthesis**

Clavulanic acid (C06662) is a substance that functions as β-lactamase inhibitor [4]. β-lactamase is an enzyme which promotes the inactivation of β-lactam antibiotics that contain penicillin system or cephalosporin system. Okisapenamu skeleton is the basic skeleton (Fig. 7), and is made from L-arginine (C00062) and D-glyceraldehyde 3-phosphate (C00118). All the eight compounds in the map were correctly disassembled (Fig. 8, the entire prediction result is described in Fig. S-6).

**map00332 Carbapenem biosynthesis**

Carbapenem is classified into β-lactam antibiotics. Carbapenem skeleton (Fig 9) is made from L-proline (C00148) and malonyl-CoA (C00083) or L-glutamate (C00025). Fig 10 is the disassembly of MM 4550, as an example. Our proposed workflow successfully disassembled 22 out of 23 molecules (the entire prediction result is described in Fig. S-7).

**map00333 Prodigiosin biosynthesis**

Prodigiosizwn is a group of natural antibiotics with three pyrrole rings, and part of their biosynthetic pathway has been revealed [5]. In the middle of the pathway, 4-methoxy-2,2'-bipyrrole-5-carbaldehyde (C21570) is generated from L-proline (C00148) and L-serine (C00065). This molecule contains two pyrrole rings, of which the first rings are originated from L-proline, and the atoms in the second ring have three origins: part of L-proline, malonyl-CoA (C00083), and L-serine. However, these molecules were not correctly disassembled, but erroneously disassembled to two pyrrole rings originated from two L-proline molecules (Fig 11). Throughout, we admit that most of the molecules in this map were not correctly disassembled (the entire prediction result is described in Fig. S-8).

**map00401 Novobiocin biosynthesis**

Novobiocin (C05080), coumermycin A1 (C05073), and clorobiocin (C12032) are classified into coumarin antibiotics, and have been isolated from various *Streptomyces* species [6]. Structures of coumarin antibiotics consist of a coumarin moiety and L-tyrosine (C00082) moiety as the basic skeleton, as well as the additional units including D-lyxose (C00476) moiety, pyrrole ring, and the isoprene units. Among the 18 molecules in this map, 17 were correctly disassembled (Fig 12). The calculation took more than six hours and did not end for the remaining one, coumermycin A1 (the entire prediction result is described in Fig. S-9).

**map00402 Benzoxazinoid biosynthesis**

Benzoxazinoids are natural products that are used for chemical defense and allelopathy, and some biosynthetic machineries have already been revealed [7]. The biosynthesis starts from chorismic acid (C00251), goes through indole-3-glycerol-phosphate (C03506), and goes to an important intermediate, 2-hydroxy-1,4-benzoxazin-3-one (HBOA; C15769), which is a derivative of indole where an oxygen atom is inserted into its pyrrole ring. HBOA becomes a basic skeleton, and the glycosides of 2,4-dihydroxy-7-methoxy-1,4-benzoxazin-3-one (C04720), which is a benzoxazinoid (Fig. 13). Our proposed workflow correctly disassembled the intermediates from indole-3-glycerol-phosphate (C03506) to HBOA, and showed that these contain indole moieties as the basic skeleton. The molecules located after HBOA were successfully disassembled to HBOA and their modifications (hydroxyl, methyl and glycosyl groups). All six molecules could be disassembled correctly (the entire prediction result is described in Fig. S-10).

**map00403 Indole diterpene alkaloid biosynthesis**

Indole diterpene alkaloids are meroterpenoids consisting of an indole (C00463) and four isoprene (C16521) units, and are a group of molecules containing mycotoxins such paxilline (C13782) and lolitrem B (C20551). The key in this map is the pathway that synthesizes paspaline (C20530), which is an intermediate made from geranylgeranyl diphosphate (C00353) and indole-3-glycerol-phosphate (C03506). This intermediate is the source of various indole terpenes such as paxilline (C13782), aflatrem (C20555), lolitrem B (C20551), and terpendole K (C20552). It is supposed that paspaline (C20530) is synthesized by repetitive epoxidation and cyclization of digerpene moiety derived from geranylgeranyl diphosphate (C00353) [8, 9]. Paxilline is synthesized by oxidation of paspaline [8, 10, 11]. The molecules located in the pathway from paspaline to paxilline were disassembled into appropriate units. The four paxilline derivatives, such as penitrem D (C20596), were also appropriately disassembled into paxilline and modifications (oxidation and isoprenylation). Aflatrem was also basically the same, since its pathway is similar to that of paxilline [10, 12]. Lolitrem B and terpendole K are epoxides, which are different from paxilline and aflatrem. However, they are very similar in terms of their pathways, and are synthesized by the oxidation and isoprenylation of paspaline [13, 14]. To sum up, all the 30 indole diterpene alkaloids were correctly disassembled (Fig 14, the entire prediction result is described in Fig. S-11).

**map00404 Staurosporine biosynthesis**

This map contains the pathway from L-tryptophan (C00078) to staurosporine (C02079), violacein (C21136), rebeccamycin (C19701), pyrrolnitrin (C12491), and fumitremorgin A (C20564). Staurosporine is a representative indolocarbazole alkaloids, and it acts as potent protein kinase inhibitors [15]. Biosynthesis of staurosporine has an important intermediate, chromopyrrolic acid (C21125), which is spontaneously synthesized by IPA imine (C21124) dimer [16]. Chromopyrrolic acid becomes an aglycone called K-252c (C21126) by aryl-aryl coupling catalyzed by cytochrome P450 [17]. The structure obtained by the removal of oxygen from K-252c corresponds to the basic indolocarbazole skeleton (Fig 15). Staurosporine is synthesized by transferring dTDP-3-amino-2,3,6-trideoxy-4-keto-D-glucose (C12318) residue and the following methylation to the aglycone. We confirmed that the aglycone was properly disassembled into the two BUs corresponding to two L-tryptophan and the nucleotide sugar moieties.

Violacein (C21136) is a natural purple pigmentation from *Chromobacterium violaceum*, and is used in medical applications, such as anti-cancer agents, antibacterial agents, and antiviral agents [16]. In the biosynthesis of violacein and deoxyviolacein (C21133), IPA imine dimer turns into a key intermediate named protodeoxyviolaceinic acid (C21131) [16]. Thereafter, violacein and deoxyviolacein are synthesized by the hydroxylation of the indole ring [16]. We confirmed that the six related molecules were correctly disassembled, showin that the center pyrrole ring is originated from two separate L-tryptophan molecules.

Rebeccamycin has an indolocarbazole skeleton, as with staurosporine. First, dichloro-chromopyrrolic acid (C19698) is synthesized from 7-chloro-L-tryptophan (7-CLT, C19687), followed by the production of the aglycone of rebeccamycin [18]. Both were able to correctly disassembled. Pyrrolnitrin is a natural product derived from L-tryptophan, and have potent antibacterial activity [19]. In the biosynthesis pathway, the donor of the reaction from 7-CLT to monodechloroaminopyrrolnitrin (MDA, C21110) is unclear, and the ring structure is greatly changed during the reaction, therefore we defined MDA as a BU. The molecules located between MDA and pyrrolnitrin were correctly disassembled. Fumitremorgin molecules have brevianamide F (C20563) as the basic skeleton consisting of L-tryptophan and L-proline moieties, and are synthesized by multiple isoprenylation, hydroxylation, and methylation. The disassembling of the relevant nine molecules was successful (Fig 16). To sum up, all the 32 molecules in this metabolic map were disassembled successfully (the entire prediction result is described in Fig. S-12).

**map00405 Phenazine biosynthesis**

Phenazine (C21476) has a symmetry structure, and is made from two molecules of chorismic acid (C00251) [20]. We defined (1R,10aS)-1,4,10,10a-tetrahydrophenazine-1-carboxylate (C21411) as a BBU, which enabled to detect its derivatives. Thus, it was made possible to perform correctly disassembled for almost all molecules. On the other hand, the two anthranilic acid (C00108) derivatives having quinolone rings, which are synthesized after the ring closure by substituting the C_8_ unit from octanoyl-CoA (C01944), were not disassembled correctly. To sum up, 22 molecules in this map were successfully disassembled (Fig. 17), and the other three were not (the entire prediction result is described in Fig. S-13).

**map00521 Streptomycin biosynthesis**

Streptomycin is aminocyclitol-aminoglycoside antibiotic generated by *Streptomyces griseus*. Streptomycin (C00413) consists of three parts, which are all synthesized from D-glucose [21]. The streptidine substructure is synthesized by substitution of an amidine group from L-arginine and phosphate group from ATP on D-glucose. The remaining two substructures are the streptose (6-deoxyhexose) moiety and an N-methyl-L-glucosamine moiety, both of which are from D-glucose. All the 15 molecules in this map were correctly disassembled (Fig 18, the entire prediction result is described in Fig. S-14).

**map00524 Neomycin, kanamycin and gentamicin biosynthesis**

The molecules in this map are classified into aminoglycoside antibiotics. Neomycins are synthesized via an intermediate named paromamine (C01743), which consists of an amino saccharide from D-glucose and UDP-N-acetyl-α-D-glucosamine [22]. In addition to this intermediate, D-ribose as another amino sugar is BUs. Gentamicins are also biosynthesized through paromamine [22]. In addition to this intermediate, amino sugars derived from D-xylose is a BU. Kanamycins are synthesized in two pathways, the one via paromamine, and the other via UDP-D-kanosamine (C12211) and an amino sugar from D-Glucose [22]. The BUs are three amino sugars. Although apramycin (C01555) and oxyapramycin (C17997) were not correctly disassembled, the other 67 molecules were successfully disassembled (Fig. 19, the entire prediction result is described in Fig. S-15 and S-16).

**map00525 Acarbose and validamycin biosynthesis**

Acarbose (C06802) and validamycin A (C12112) are used as antidiabetic agents and antifungal antibiotics, respectively [21]. Both have C_7_N moiety derived from 2-epi-5-epi-valiolone (C17692) [21]. All the 29 compounds in this map were correctly disassembled (Fig. 21, the entire prediction result is described in Fig. S-17).

**map00901 Indole alkaloid biosynthesis**

Ergot alkaloids include fumigaclavine C (C20438) and agroclavine (C09023), and they have an ergoline skeleton (Fig. 21) and are important pharmaceuticals that are producible in biologically industrial scale [23]. The starting material is L-tryptophan, and it generates an ergoline skeleton by isoprenylation and ring closure reaction. Psilocybin (C07576) and psilocin (C08312) are tryptamine alkaloids contained in magic mushrooms, and enzymes in the biosynthesis are already revealed [24]. Vinblastine (C07201) and vincristine (C07204) are medically important bisindole alkaloids, and are synthesized from tabersonine (C09244) and catharanthine (C09107), respectively [25]. Among the molecules in this map, 3-α-(S)-strictosidine (C03470) could be correctly disassembled, whereas the other 55 molecules were correctly disassembled (Fig. 22, the entire prediction result is described in Fig. S-18 and S-19).

**map00940 Phenylpropanoid biosynthesis**

Phenylpropanoids are plant-derived natural products involved in almost all plant responses for biological or abiotic stimuli [26], and are originated from L-phenylalanine and L-tyrosine. The C_6_C_3_ unit (the phenylpropane unit) is a basic skeleton. Among these molecules, 51 were correctly disassembled (Fig. 23), although the other four molecules were not. First, 3-(2-Carboxyethenyl)-cis,cis-muconate (C04366) is synthesized by the oxidative cleavage of caffeic acid (C01197). Since this cleavage is a special reaction in this map, it is difficult to prepare as a BU derived from L-phenylalanine. The remaining three were the three coumarin molecules. These are generated by a ring formation by intramolecular condensation. A problem with aromaticity prevented the matching with a prepared C_6_C_3_ unit. (the entire prediction result is described in Fig. S-20).

**map00941 Flavonoid biosynthesis**

Flavonoid is a generic term for natural products biosynthesized from cinnamoyl-CoA (C00540) and p-coumaroyl-CoA (C00223). Biosynthetic pathway goes on to such as anthocyanins and isoflavonoids. A C_6_C_3_ unit of cinnamoyl-CoA and p-coumaroyl-CoA is subjected to the elongation of the carbon chain by malonyl-CoA, and the following cyclization reaction yields chalcone a basic skeleton of flavonoid (Fig. 24). As an example, pinocembrin (C09827) has been found to be synthesized from cinnamoyl-CoA and malonyl-CoA [27]. Here we defined benzenetriol as a BU. Pinocembrin was disassembled into two, a benzenetriol moiety and a cinnamoyl-CoA moiety (Fig 25). Similarly, benzenediol was also be defined as a biosynthesis unit, which contributed to the enhancement of the correct answer rate and to efficient calculation time. Thus, all the 63 molecules were correctly disassembled (the entire prediction result is described in Fig. S-21).

**map00942 Anthocyanin biosynthesis**

Anthocyanins are common plant pigments, and their basic skeleton consists of 2-phenyl benzopyrylium unit (Fig. 26) originated from pelargonidin (C05904), cyanidin (C05905), and delphinidin (C05908). Their glycoside units are glucose, rhamnose, and glucuronate, and they are originated from the relevant UDP sugars. Other units come from p-coumaroyl-CoA and malonyl-CoA. Biosynthetic pathways of some molecules are not yet clarified, however, estimated from the biosynthetic machineries of other derivatives, all 63 molecules were thought to be correctly disassembled (Fig 27, the entire prediction result is described in Fig. S-22, S-23 and S-24).

**map00943 Isoflavonoid biosynthesis**

Isoflavonoids are contained in plant estrogens, and have isoflavone skeleton (Fig. 28). Their starting materials are liquiritigenin (C09762) and naringenin (C00509), both of which have a flavone skeleton (Fig. 28), not isoflavone skeleton. Isoflavone skeleton is generated by the rearrangement reaction of the phenyl group in the flavone skeleton [28]. Isoflavone derivatives are synthesized by methylation, hydroxylation, and isoprenylation, and the ring closure. Of 59 molecules in this map, 57 were successfully disassembled (Fig 29, the entire prediction result is described in Fig. S-25).

**map00944 Flavone and flavonol biosynthesis**

Flavons and flavonols (Fig. 30) are common flavonoids in the plant kingdom. Apigenin (C01477) and kaempferol (C05903) are their starting materials, respectively. Their BUs are very easy to find, and all 49 molecules were correctly disassembled (Fig. 31, the entire prediction result is described in Fig. S-26).

**map00945 Stilbenoid, diarylheptanoid and gingerol biosynthesis**

Stilbenoids, diarylheptanoids, and gingerol are groups of phenolic compounds, and are biosynthesized from cinnamic acid or p-coumaroyl-CoA. Among stilbenoids, resveratrol (C03582) is famous as the content of in grapes and its cancer preventive activity [29]. Among diarylheptanoids, curcumin (C10443) is included in turmeric and used in dietary supplements and cosmetics [30]. Gingerol is pungent component contained in ginger. All their molecules were correctly disassembled (Fig 32, the entire prediction result is described in Fig. S-27).

**map00950 Isoquinoline alkaloid biosynthesis**

Isoquinoline alkaloids have an isoquinoline skeleton from L-tyrosine (Fig 33). (S)-reticuline (C02105) is an important precursor of various benzyl isoquinoline alkaloids [31, 32]. By checking the conversion pattern of chemical structures, all 98 molecules were confirmed to be correctly disassembled to be verified, it was confirmed that was able to correctly predicted (Fig 34, the entire prediction result is described in Fig. S-28 and S-29).

**map00960 Tropane, piperidine and pyridine alkaloid biosynthesis**

Tropanes have two piperidine (C01746) moieties connected by a bridge [33]. Although detailed biosynthetic machinery is not yet clear, BUs contain 1-methylpyrrolinium (C06178) moiety and acetone moiety. Most tropane alkaloids were correctly disassembled. Piperidine alkaloids and pyridine alkaloids are biosynthesized from L-lysine and nicotinic acid. Of 58 molecules, 47 were correctly disassembled (Fig. 35). However, many of those biosynthetic machineries have not been clear, and many molecules were unable to be disassembled (the entire prediction result is described in Fig. S-30).

**map00965 Betalain biosynthesis**

Betalains are nitrogen-containing pigments, and they are synthesized on the basis of L-tyrosine and betalamic acid (C08538) [34]. Lampranthin II (C08552) and celosianin II (C08542) contains ferulic acid (C01494) moiety originated from 1-O-feruloyl-β-D-glucose (C17759). However, instead of ferulic acid, they were disassembled into p-coumaric acid and methyl and hydroxy groups. This is because ferulic acid is originated from p-coumaric acid, and ferulic acid is not in BUL. We regarded them to be correct. Thus, all the 20 molecules were correctly disassembled (Fig. 36, the entire prediction result is described in Fig. S-31).

**map00966 Glucosinolate biosynthesis**

Glucosinolates are sulfur-containing natural products present in cruciferous and capparaceae plants. Every glucosinolate is originated from the relevant α-amino acid as the starting material, and has beta-thio-glucose moiety and sulfonated oxime moiety. We prepared BU to properly disassemble glucosinolates as the end products of this map. The path from L-methionine is subjected to carbon chain extensions and generates glucosinolates with different carbon chain lengths. All 53 molecules were correctly disassembled (Fig. 37, the entire prediction result is described in Fig. S-32 and S-33).

**map01058 Acridone alkaloid biosynthesis**

Acridone alkaloids have an acridine skeleton and are synthesized from anthranilic acid (C00108) [35]. The biosynthetic machinery of these molecules is not clear. Therefore, we regarded that these molecules are synthesized from the acridine skeleton (Fig. 38) as the BU, followed by hydroxylation, methylation, and isoprenylation. All 15 molecules were correctly disassembled (Fig. 39, the entire prediction result is described in Fig. S-34).

**References**

1. Tercero JA, Espinosa JC, Lacalle RA, Jiménez A. The biosynthetic pathway of the aminonucleoside antibiotic puromycin, as deduced from the molecular analysis of the pur cluster of Streptomyces alboniger. J Biol Chem. 1996;271:1579–90.
2. Gaudelli NM, Long DH, Townsend CA. β-Lactam formation by a non-ribosomal peptide synthetase during antibiotic biosynthesis. Nature. 2015;520:383–7.
3. Brakhage AA. Molecular regulation of β-lactam biosynthesis in Filamentous fungi. Microbiol Mol Biol Rev. 1998;62:547–85.
4. Reading C, Cole M. Clavulanic acid: a beta-lactamase-inhiting beta-lactam from Streptomyces clavuligerus. Antimicrob Agents Chemother. 1977;11:852–7.
5. Williamson NR, Fineran PC, Leeper FJ, Salmond GPC. The biosynthesis and regulation of bacterial prodiginines. Nat Rev Microbiol. 2006;4:887–99.
6. Chen H, Walsh CT. Coumarin formation in novobiocin biosynthesis: β-hydroxylation of the aminoacyl enzyme tyrosyl-S-NovH by a cytochrome P450 NovI. Chem Biol. 2001;8:301–12.
7. Frey M, Schullehner K, Dick R, Fiesselmann A, Gierl A. Benzoxazinoid biosynthesis, a model for evolution of secondary metabolic pathways in plants. Phytochemistry. 2009;70:1645–51.
8. Tagami K, Liu C, Minami A, Noike M, Isaka T, Fueki S, et al. Reconstitution of biosynthetic machinery for indole-diterpene paxilline in Aspergillus oryzae. J Am Chem Soc. 2013;135:1260–3.
9. Saikia S, Parker EJ, Koulman A, Scott B. Four gene products are required for the fungal synthesis of the indole-diterpene, paspaline. FEBS Lett. 2006;580:1625–30.
10. Tagami K, Minami A, Fujii R, Liu C, Tanaka M, Gomi K, et al. Rapid reconstitution of biosynthetic machinery for fungal metabolites in Aspergillus oryzae: total biosynthesis of aflatrem. ChemBioChem. 2014;15:2076–80.
11. Saikia S, Parker EJ, Koulman A, Scott B. Defining paxilline biosynthesis in Penicillium paxilli: Functional characterization of two cytochrome P450 monooxygenases. J Biol Chem. 2007;282:16829–37.
12. Nicholson MJ, Koulman A, Monahan BJ, Pritchard BL, Payne GA, Scott B. Identification of two aflatrem biosynthesis gene loci in Aspergillus flavus and metabolic engineering of Penicillium paxilli to elucidate their function. Appl Environ Microbiol. 2009;75:7469–81.
13. Saikia S, Takemoto D, Tapper BA, Lane GA, Fraser K, Scott B. Functional analysis of an indole-diterpene gene cluster for lolitrem B biosynthesis in the grass endosymbiont Epichloë festucae. FEBS Lett. 2012;586:2563–9.
14. Motoyama T, Hayashi T, Hirota H, Ueki M, Osada H. Terpendole E, a kinesin Eg5 inhibitor, is a key biosynthetic intermediate of indole-diterpenes in the producing fungus Chaunopycnis alba. Chem Biol. 2012;19:1611–9.
15. Omura S, Sasaki Y, Iwai Y, Takeshima H. Staurosporine, a potentially important gift from a microorganism. J Antibiot. 1995;48:535–48.
16. Füller JJ, Röpke R, Krausze J, Rennhack KE, Daniel NP, Blankenfeldt W, et al. Biosynthesis of violacein, structure and function of l-tryptophan oxidase VioA from Chromobacterium violaceum. J Biol Chem. 2016;291:20068–84.
17. Howard-Jones AR, Walsh CT. Nonenzymatic oxidative steps accompanying action of the cytochrome P450 enzymes StaP and RebP in the biosynthesis of staurosporine and rebeccamycin. J Am Chem Soc. 2007;129:11016–7.
18. Spolitak T, Ballou DP. Evidence for catalytic intermediates involved in generating the chromopyrrolic acid scaffold of rebeccamycin by RebO and RebD. Arch Biochem Biophys. 2015;573:111–9.
19. Kirner S, Hammer PE, Hill DS, Altmann A, Fischer I, Weislo LJ, et al. Functions encoded by pyrrolnitrin biosynthetic genes from Pseudomonas fluorescens. J Bacteriol. 1998;180:1939–43.
20. Mentel M, Ahuja EG, Mavrodi DV, Breinbauer R, Thomashow LS, Blankenfeldt W. Of two make one: the biosynthesis of phenazines. ChemBioChem. 2009;10:2295–304.
21. Flatt PM, Mahmud T. Biosynthesis of aminocyclitol-aminoglycoside antibiotics and related compounds. Nat Prod Rep. 2007;24:358–92.
22. Kudo F, Eguchi T. Biosynthetic genes for aminoglycoside antibiotics. J Antibiot. 2009;62:471–81.
23. Wallwey C, Matuschek M, Xie XL, Li SM. Ergot alkaloid biosynthesis in Aspergillus fumigatus: Conversion of chanoclavine-I aldehyde to festuclavine by the festuclavine synthase FgaFS in the presence of the old yellow enzyme FgaOx3. Org Biomol Chem. 2010;8:3500–8.
24. Fricke J, Blei F, Hoffmeister D. Enzymatic synthesis of psilocybin. Angew Chemie Int Ed. 2017;56:12352–5.
25. Schröder G, Unterbusch E, Kaltenbach M, Schmidt J, Strack D, De Luca V, et al. Light-induced cytochrome P450-dependent enzyme in indole alkaloid biosynthesis: tabersonine 16-hydroxylase. FEBS Lett. 1999;458:97–102.
26. Vogt T. Phenylpropanoid biosynthesis. Mol Plant. 2010;3:2–20.
27. Miyahisa I, Funa N, Ohnishi Y, Martens S, Moriguchi T, Horinouchi S. Combinatorial biosynthesis of flavones and flavonols in Escherichia coli. Appl Microbiol Biotechnol. 2006;71:53–8.
28. Dhaubhadel S, Mcgarvey BD, Williams R, Gijzen M. Isoflavonoid biosynthesis and accumulation in developing soybean seeds. Plant Mol Biol. 2003;53:733–43.
29. Jang M, Cai L, Udeani GO, Slowing KV, Thomas CF, Beecher CW, et al. Cancer chemopreventive activity of resveratrol, a natural product derived from grapes. Science. 1997;275:218–20.
30. Kita T, Imai S, Sawada H, Kumagai H, Seto H. The biosynthetic pathway of curcuminoid in turmeric (Curcuma longa) as revealed by 13C-labeled precursors. Biosci Biotechnol Biochem. 2008;72:1789–1798.
31. Facchini PJ, De Luca V. Opium poppy and Madagascar periwinkle: model non-model systems to investigate alkaloid biosynthesis in plants. Plant J. 2008;54:763–84.
32. Minami H, Kim JS, Ikezawa N, Takemura T, Katayama T, Kumagai H, et al. Microbial production of plant benzylisoquinoline alkaloids. Proc Natl Acad Sci U S A. 2008;105:7393–8.
33. Humphrey AJ, O’Hagan D. Tropane alkaloid biosynthesis. A century old problem unresolved. Nat Prod Rep. 2001;18:494–502.
34. Gandía-Herrero F, Escribano J, García-Carmona F. Betaxanthins as substrates for tyrosinase. An approach to the role of tyrosinase in the biosynthetic pathway of betalains. Plant Physiol. 2005;138:421–32.
35. Dewick PM. Medicinal natural products: a biosynthetic approach. 3rd edition. Chichester, England: John Wiley & Sons; 2009.

**Figures**


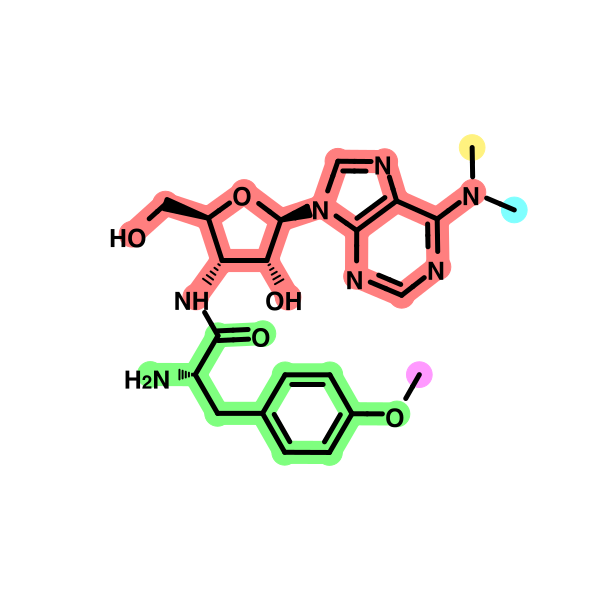


Figure 1. Disassembly of puromycin.


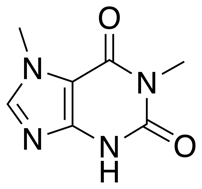


Figure 2. The basic skeleton of caffeine metabolism.


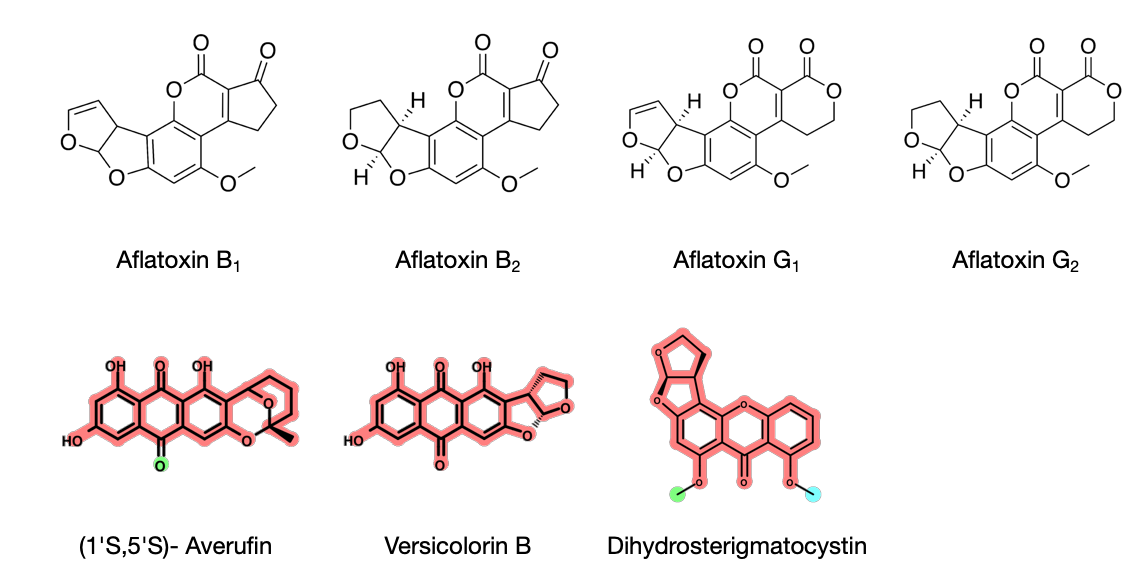


Figure 3. Disassembly of the representative molecules in map00254. Aflatoxin B_1_，Aflatoxin B_2_，Aflatoxin G_1_，Aflatoxin G_2_ were not correctly disassembled．Other molecules including (1'S,5'S)-Averufin，Versicolorin B，Dihydrosterigmatocystin were correctly disassembled.


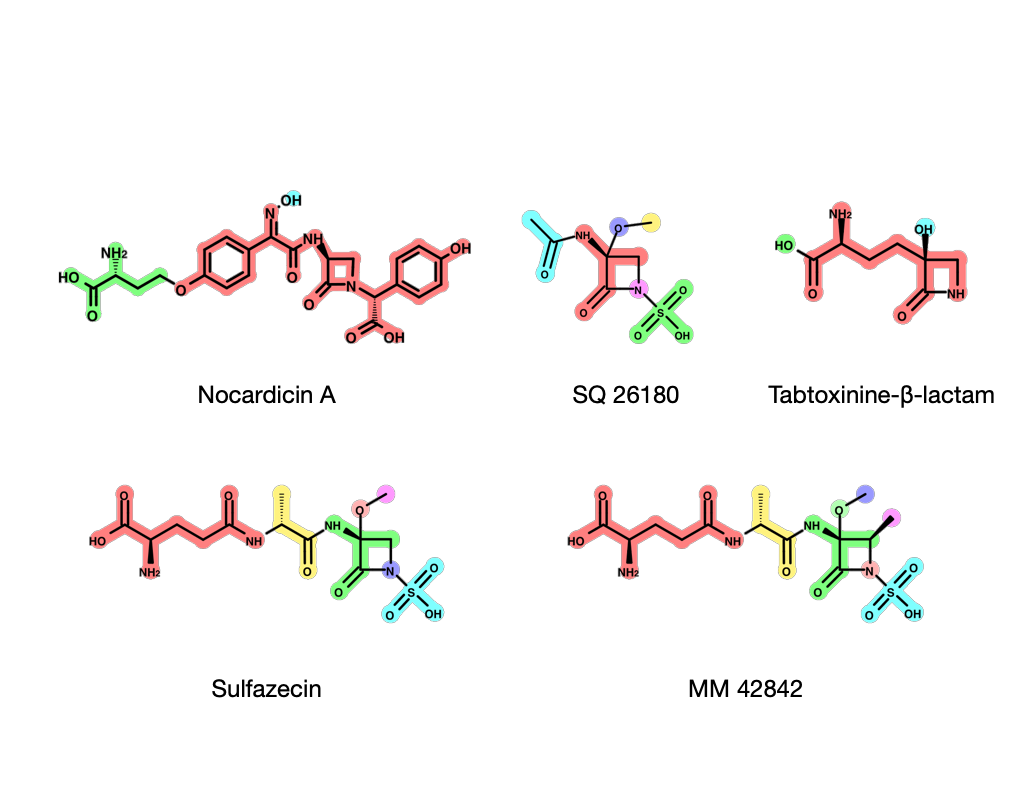


Figure 4. Disassembly of representative molecules in map00261.


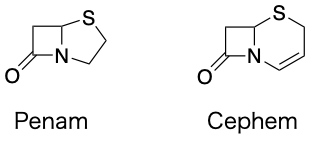


Figure 5. Penam and cephem skeletons.


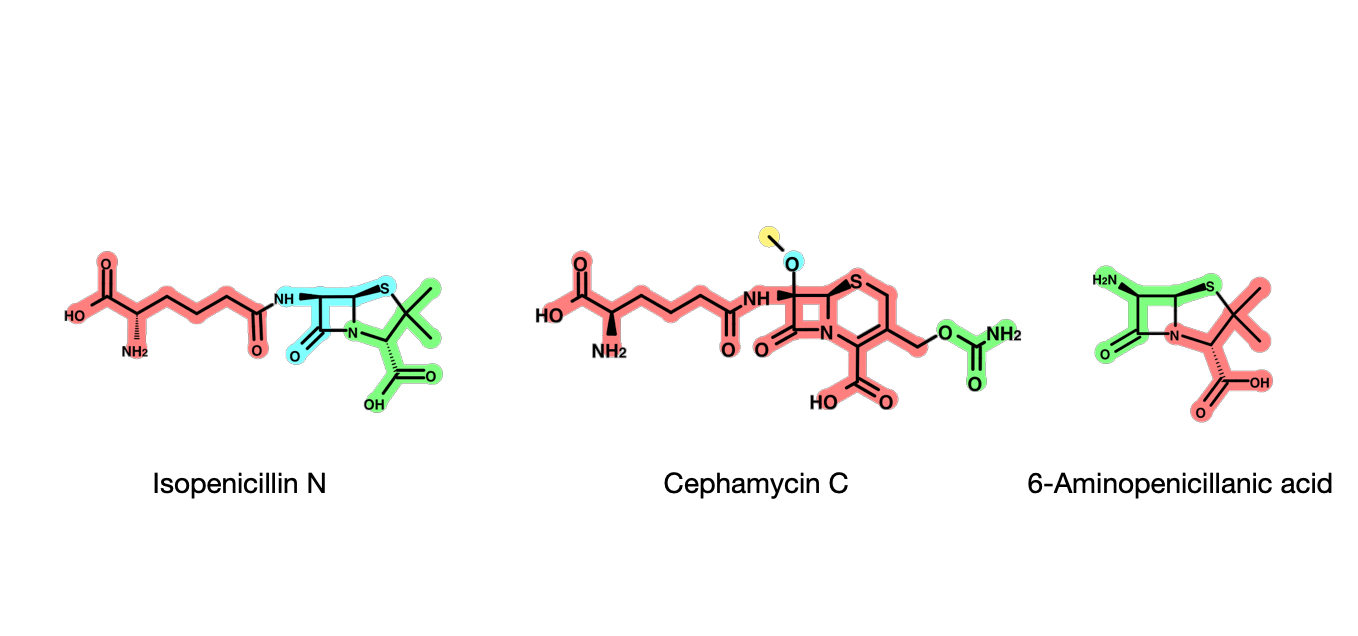


Figure 6. Disassembly of representative molecules in map00311.


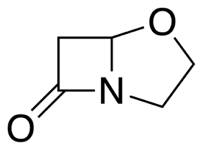


Figure 7. Okisapenamu skeleton.


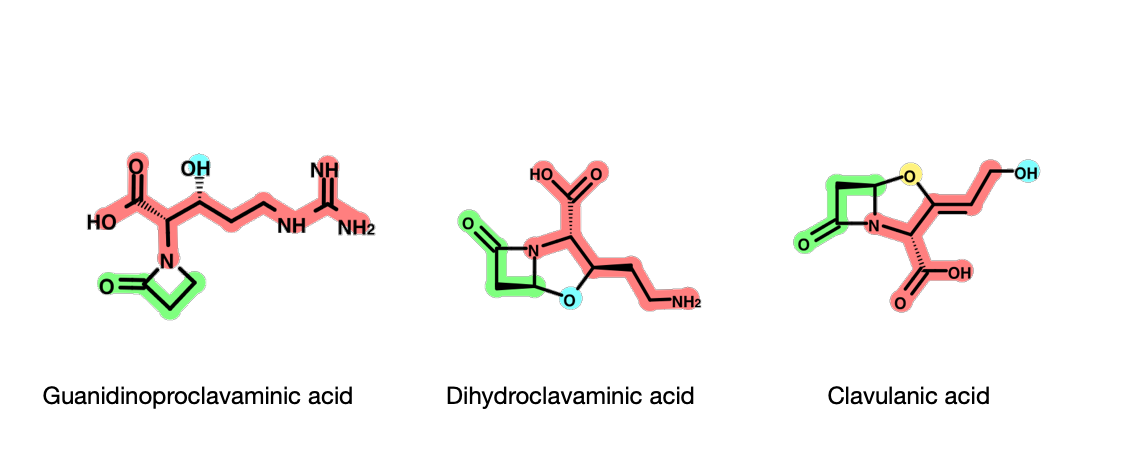


Figure 8. Disassembly of representative molecules in map00331.


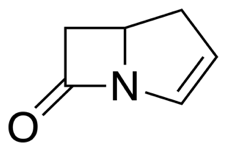


Figure 9. Carbapenem skeleton.


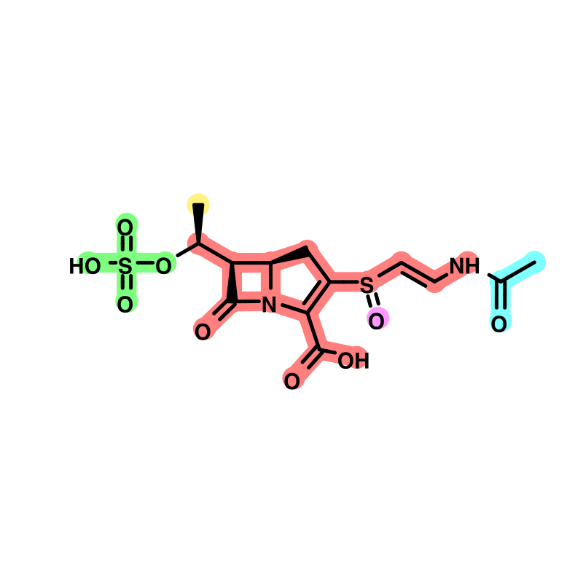


Figure 10. Disassembly of MM 455. The red highlighted substructure is an important intermediate in carbapenem biosynthesis.


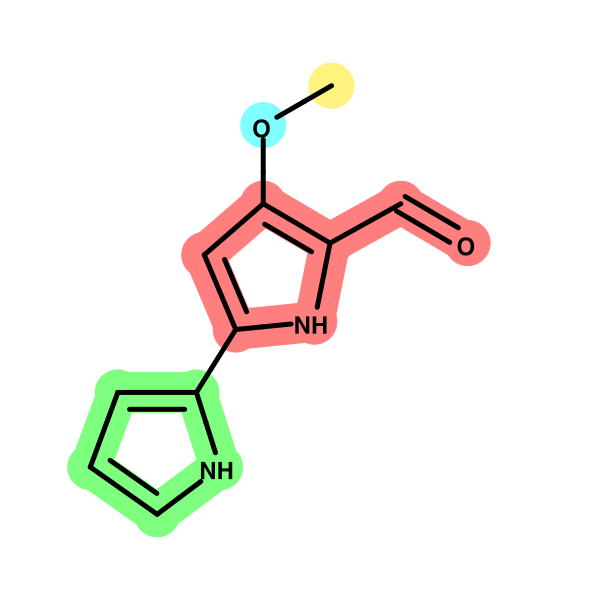


Figure 11. Incorrect disassembly of a molecule in map00333 Two pyrrole rings in 4-Methoxy-2,2'-bipyrrole-5-carbaldehyde were both regarded to be from L-proline, which is not correct.


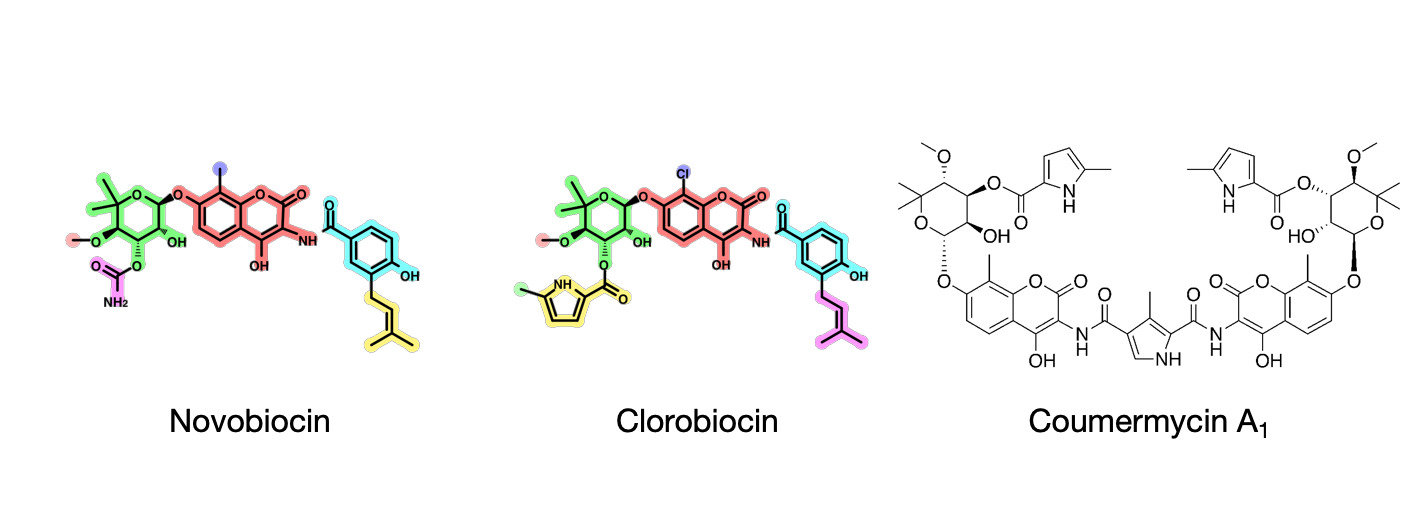


Figure 12. Disassembly of representative molecules in map00401. Coumermycin A_1_ was unable to disassemble because of the vast computational time.


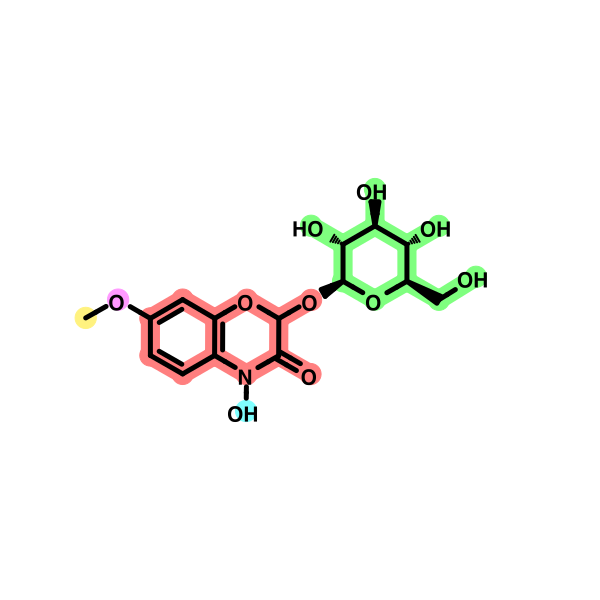


Figure 13. Disassembly of DIMBOA-glucoside.


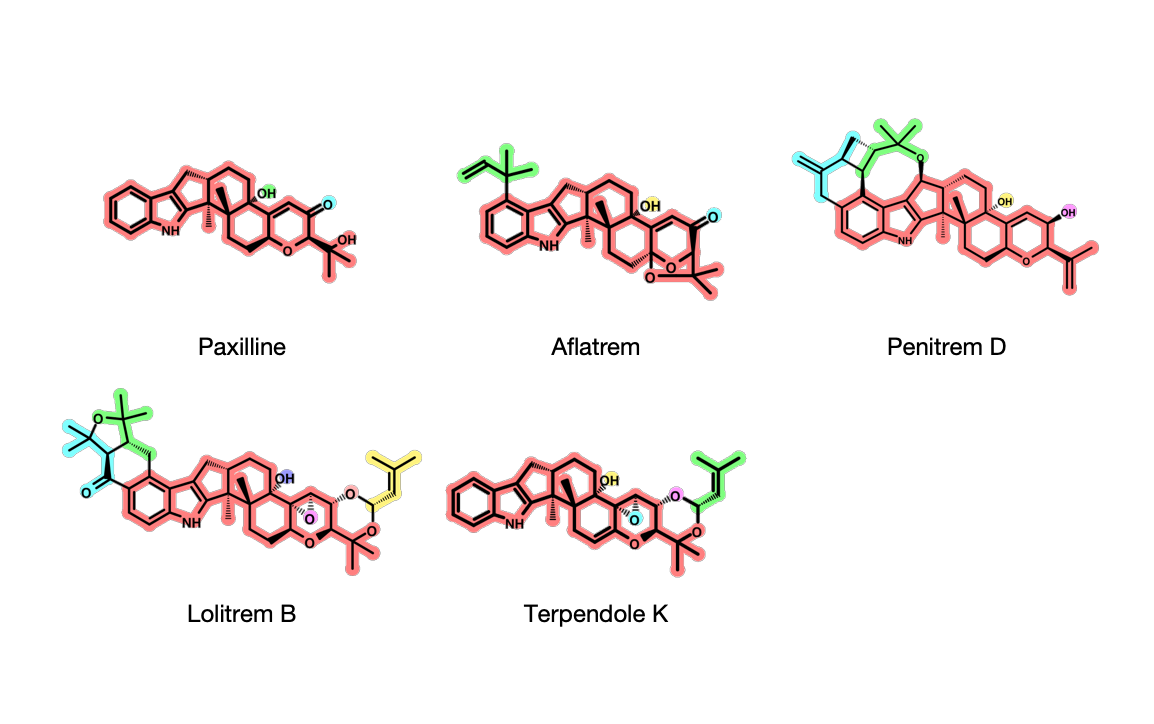


Figure 14. Disassembly of representative molecules in map00403.


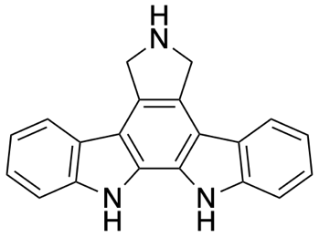


Figure 15. Indolocarbazole skeleton.


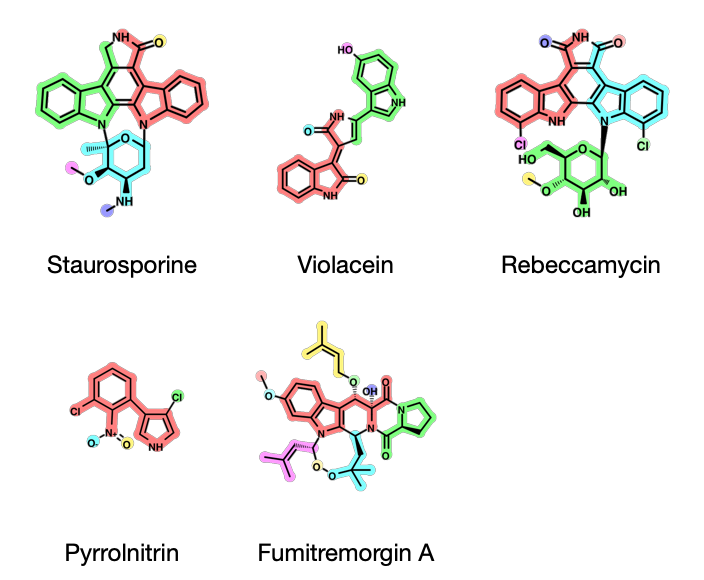


Figure 16. Disassembly of representative molecules in map00404.


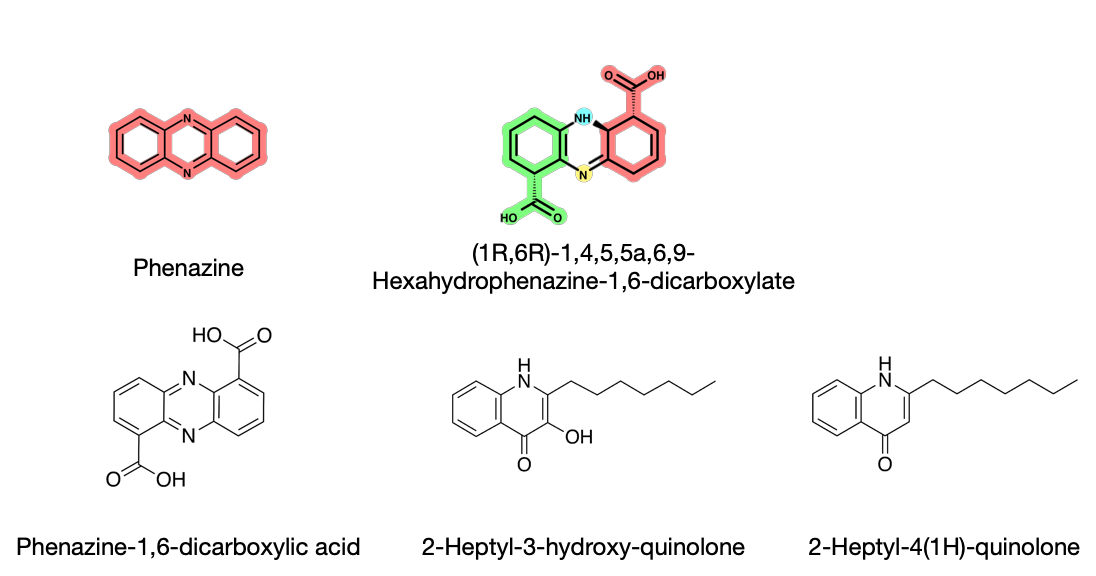


Figure 17. Disassembly of representative molecules in map00405. Phenazine-1,6-dicarboxylic acid, 2-Heptyl-3-hydroxy-quinolone, 2-Heptyl-4(1H)-quinolone were not correctly disassembled.


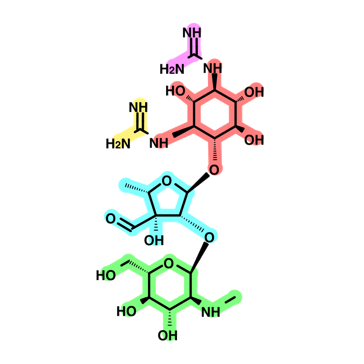


Figure 18. Disassembly of Streptomycin.


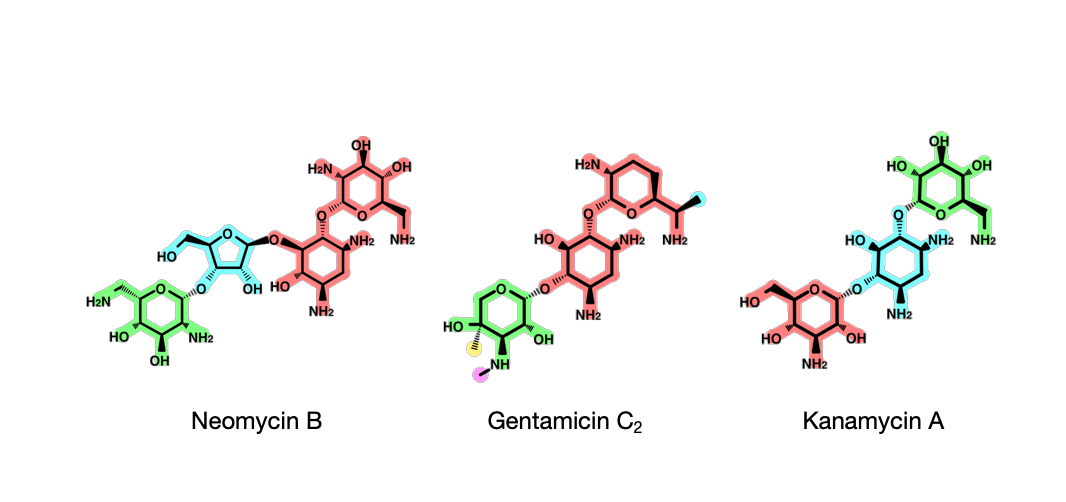


Figure 19. Disassembly of representative molecules in map00524.


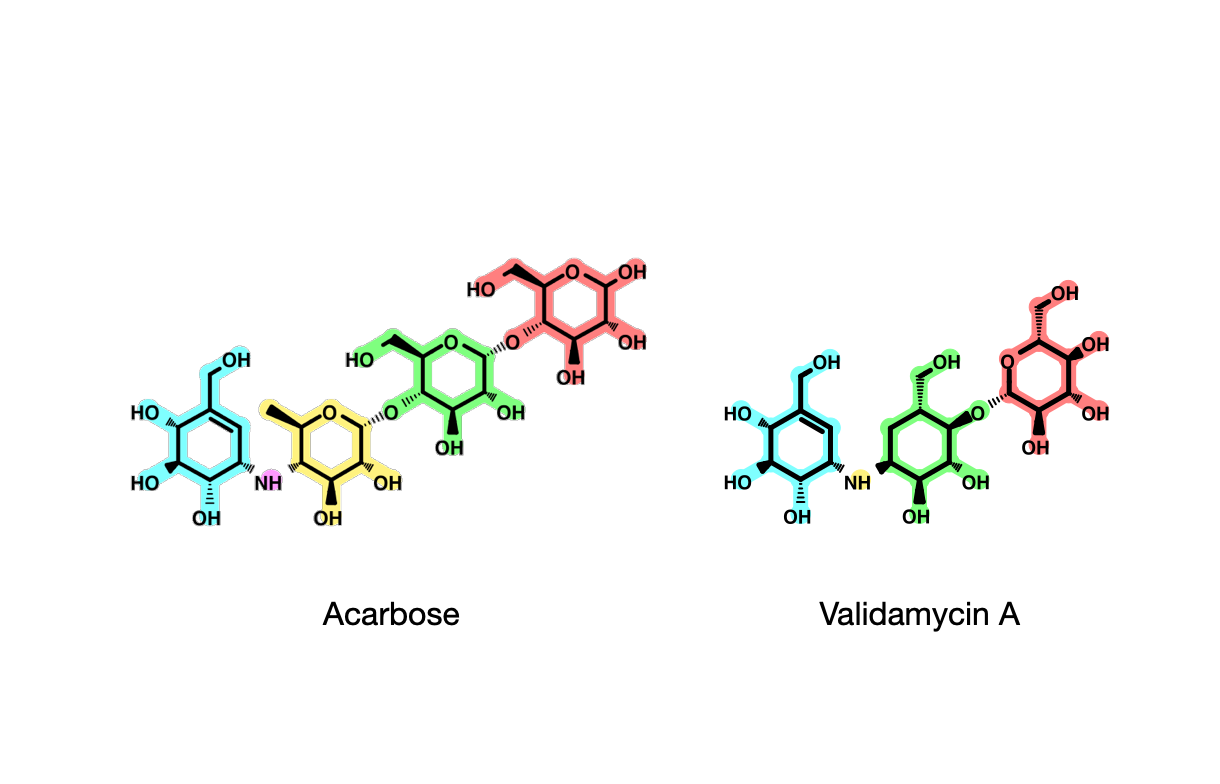


Figure 20. Disassembly of representative molecules in map00525.


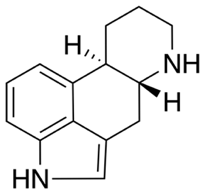


Figure 21. Ergoline skeleton.


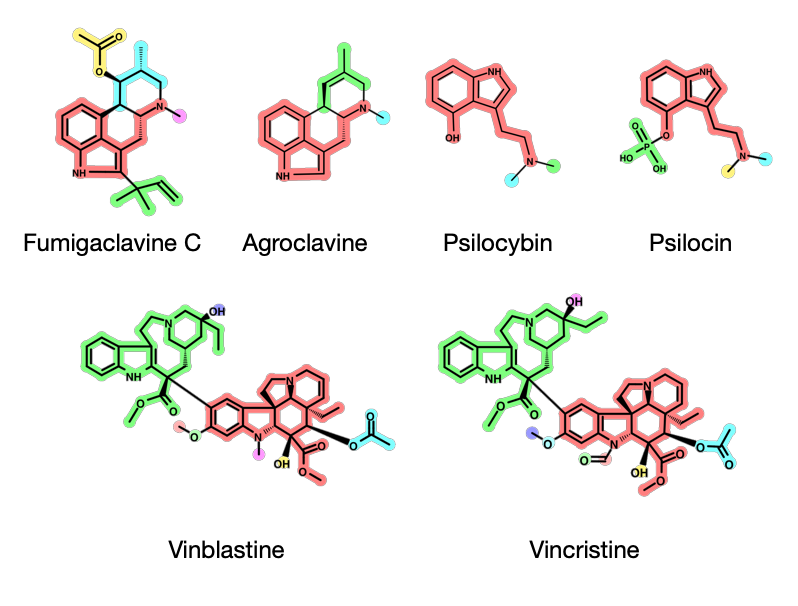


Figure 22. Disassembly of representative molecules in map00901.


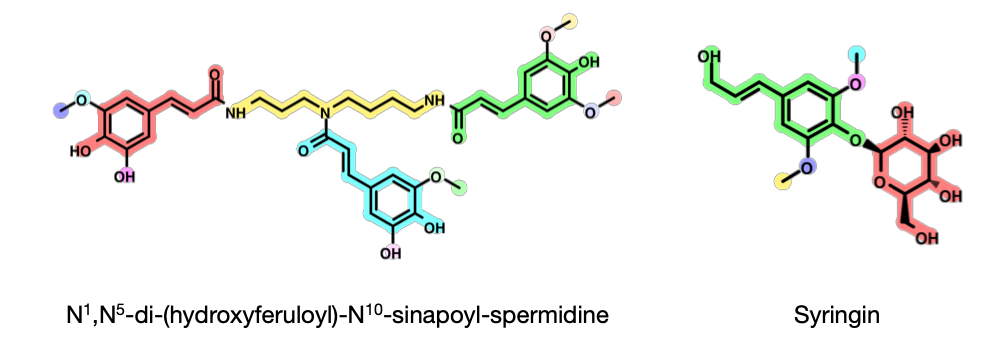


Figure 23. Disassembly of representative molecules in map00940.


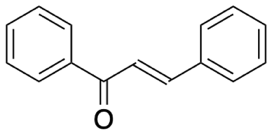


Figure 24. Chalcone.


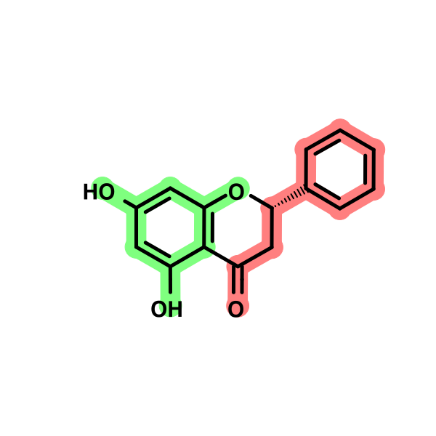


Figure 25. Disassembly of pinocembrin.


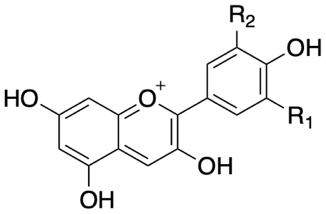


Figure 26. 2-phenyl benzopyrylium unit.


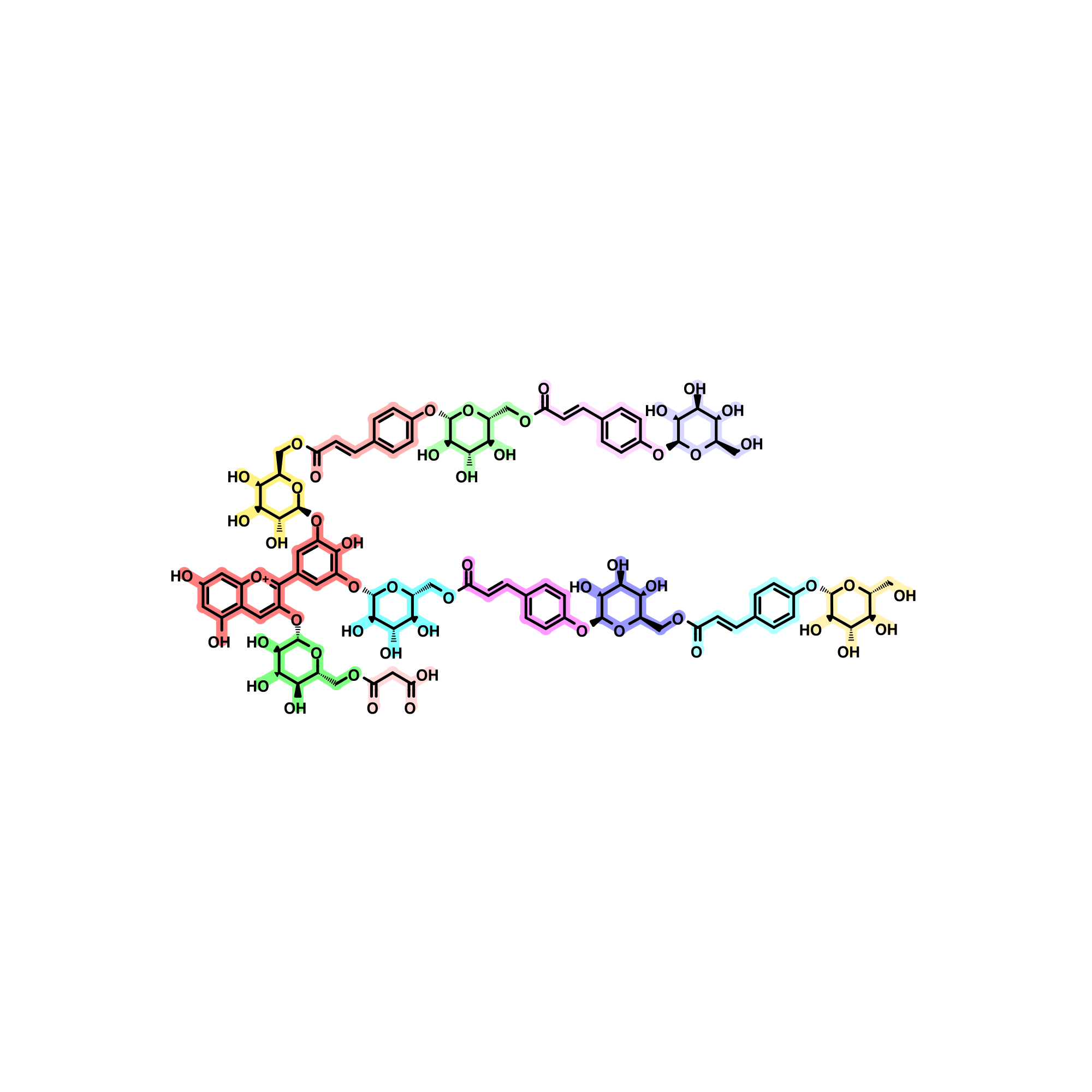


Figure 27. Disassembly of ternatin A1.


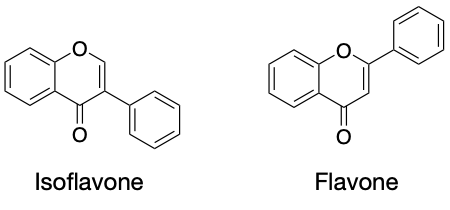


図 3‑28　Figure 28. Isoflavone and Flavone skeletons.


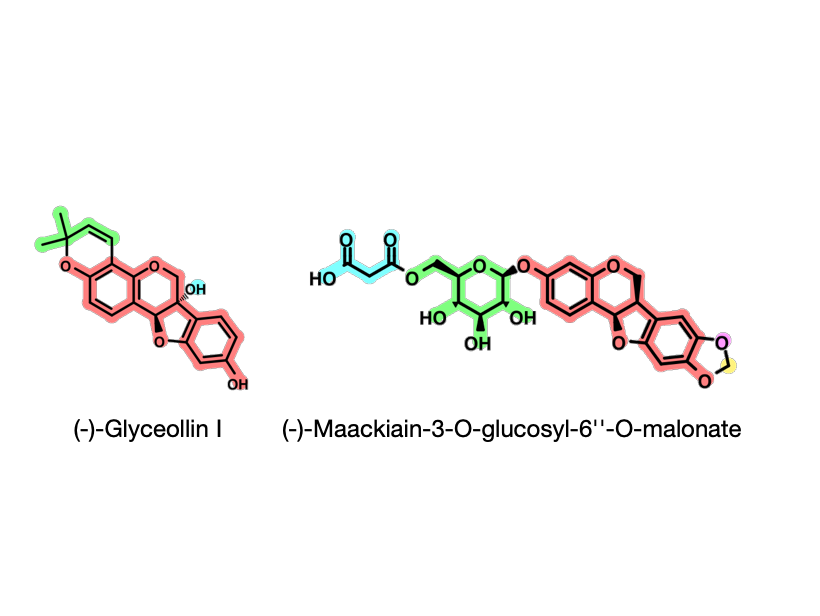


Figure 29. Disassembly of representative molecules in map00943.


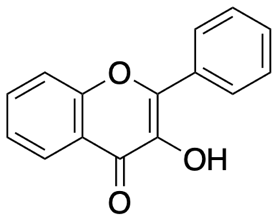


Figure 30. Flavonol.


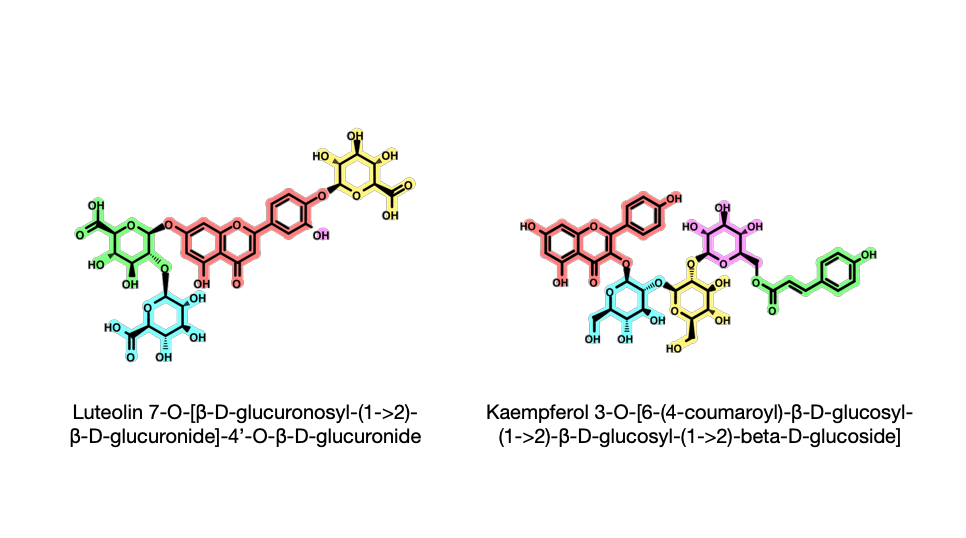


Figure 31. Disassembly of representative molecules in map00944.


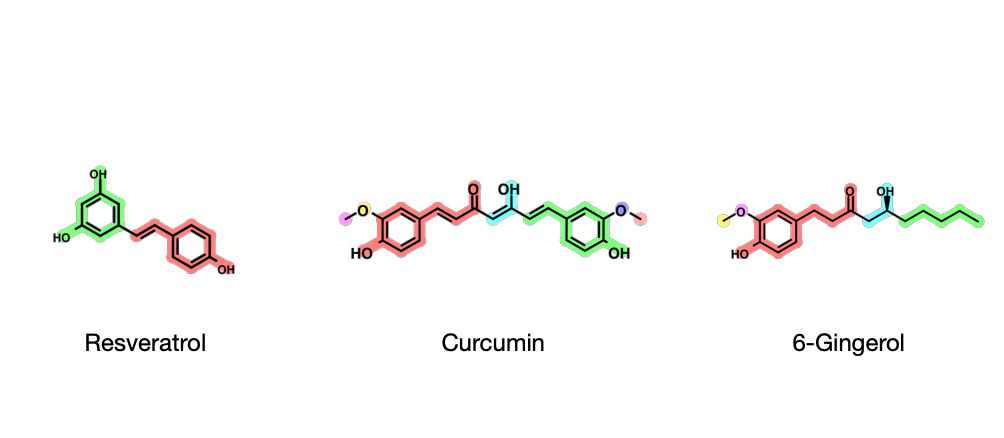


Figure 32. Disassembly of representative molecules in map00945.


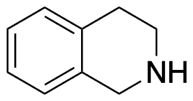


Figure 33. Isoquinoline skeleton.


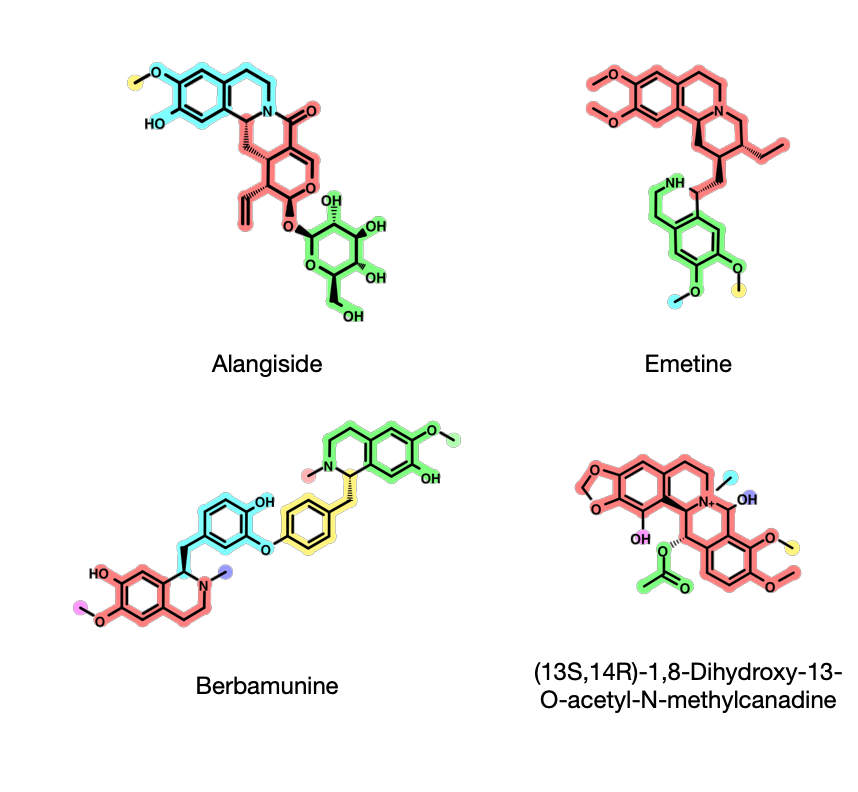


Figure 34. Disassembly of representative molecules in map00950.


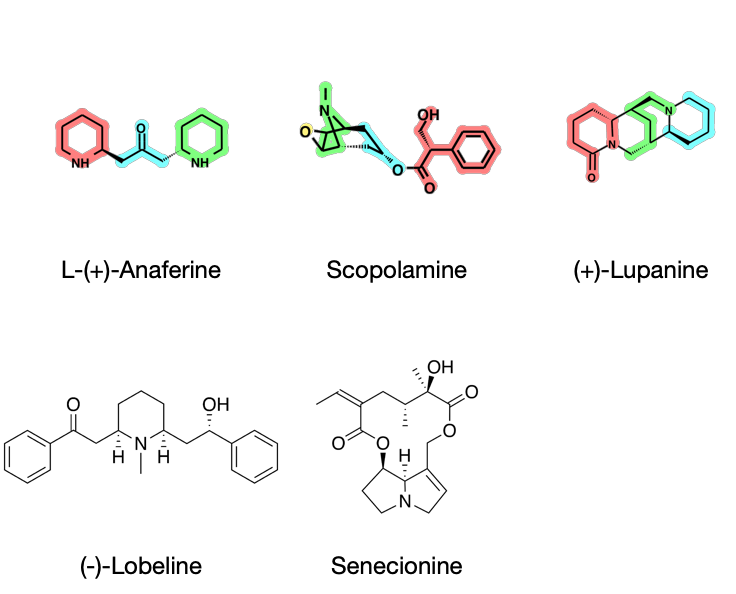


Figure 35. Disassembly of representative molecules in map00960. (-)-Lobeline and senecionine were not correctly disassembled.


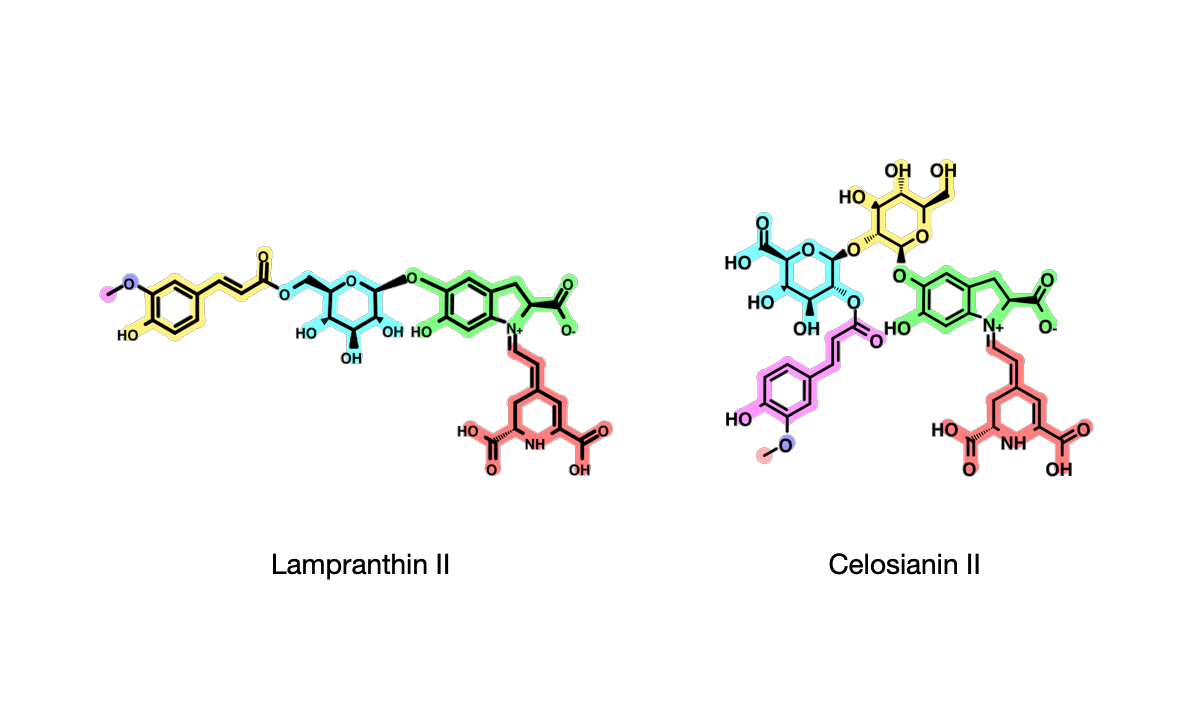


Figure 36. Disassembly of representative molecules in map00965.


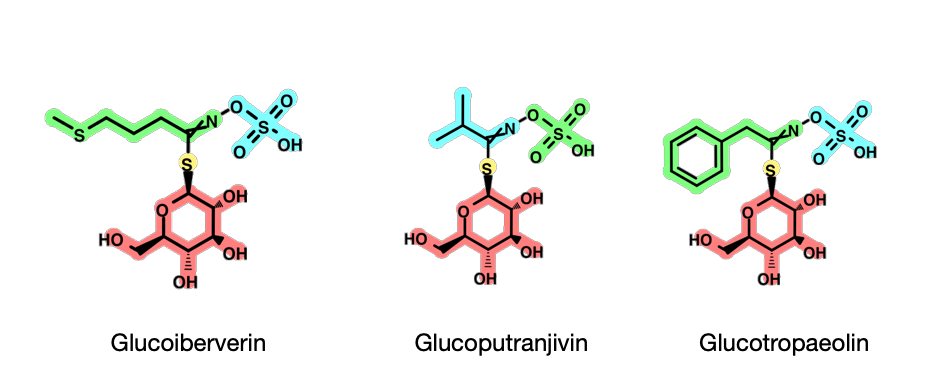


Figure 37. Disassembly of representative molecules in map00966.


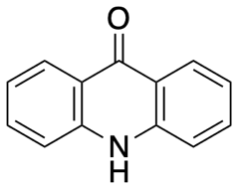


Figure 38. Acridone.


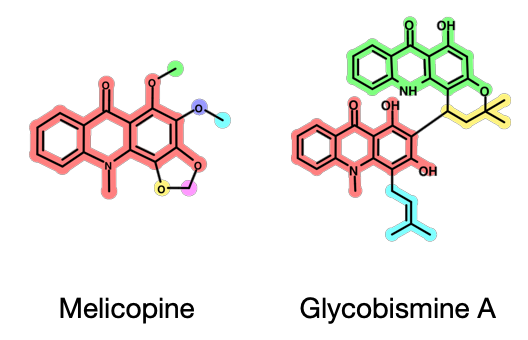


Figure 39. Disassembly of representative molecules in map01058.
